# Identification of Rice LncRNAs and Their Roles in the Rice Blast Resistance Network Using Transcriptome and Translatome

**DOI:** 10.3390/plants14172752

**Published:** 2025-09-03

**Authors:** Xiaoliang Shan, Shengge Xia, Long Peng, Cheng Tang, Shentong Tao, Ayesha Baig, Hongwei Zhao

**Affiliations:** 1 State Key Laboratory of Agricultural and Forestry Biosecurity, College of Plant Protection, Nanjing Agricultural University, Nanjing 210095, China; 2021202037@stu.njau.edu.cn (X.S.); xiashg2024@lzu.edu.cn (S.X.); 2021202038@stu.njau.edu.cn (C.T.); t2022077@njau.edu.cn (S.T.); 2 Ministry of Education Key Laboratory of Cell Activities and Stress Adaptations, School of Life Sciences, Lanzhou University, Lanzhou 730000, China; 3 State Key Laboratory of Tree Genetics and Breeding, Chinese Academy of Forestry, Beijing 100091, China; penglong2018@caf.ac.cn; 4 Research Institute of Subtropical Forestry, Chinese Academy of Forestry, Hangzhou 311400, China; 5 Department of Biotechnology, COMSATS University Islamabad Abbottabad Campus, Abbottabad 22060, Pakistan

**Keywords:** long non-coding RNAs, *Magnaporthe oryzae*, plant immunity, ceRNA, WGCNA, hormone signaling

## Abstract

Long non-coding RNAs (lncRNAs) have emerged as pivotal regulators in plant immune responses, yet their roles in rice resistance against *Magnaporthe oryzae* (*M. oryzae*) remain inadequately explored. In this study, we integrated translatome data with conventional genome annotations to construct an optimized protein-coding dataset. Subsequently, we developed a robust pipeline (“RiceLncRNA”) for the accurate identification of rice lncRNAs. Using strand-specific RNA-sequencing (ssRNA-seq) data from the resistant (IR25), susceptible (LTH), and Nipponbare (NPB) varieties under *M. oryzae* infection, we identified 9003 high-confidence lncRNAs, significantly improving identification accuracy over traditional methods. Among the differentially expressed lncRNAs (DELs), those unique to IR25 were enriched in the biosynthetic pathways of phenylalanine, tyrosine, and tryptophan, which suggests that they are associated with the production of salicylic acid (SA) and auxin (IAA) precursors, which may be involved in defense responses. Conversely, DELs specific to LTH primarily clustered within carbon metabolism pathways, indicating a metabolic reprogramming mechanism. Notably, 21 DELs responded concurrently in both IR25 and LTH at 12 h and 24 h post-inoculation, indicating a synergistic regulation of jasmonic acid (JA) and ethylene (ET) signaling while partially suppressing IAA pathways. Weighted gene co-expression network analysis (WGCNA) and competing endogenous RNA (ceRNA) network analysis revealed that key lncRNAs (e.g., LncRNA.9497.1) may function as miRNA “sponges”, potentially influencing the expression of receptor-like kinases (RLKs), resistance (R) proteins, and hormone signaling pathways. The reliability of these findings was confirmed through qRT-PCR and cloning experiments. In summary, our study provides an optimized rice lncRNA annotation framework and reveals the mechanism by which lncRNAs enhance rice blast resistance through the regulation of hormone signaling pathways. These findings offer an important molecular basis for rice disease-resistant breeding.

## 1. Introduction

Long non-coding RNAs (lncRNAs) are transcripts longer than 200 nucleotides with limited or no protein-coding potential [1]. Emerging evidence indicates that lncRNAs participate in a wide range of biological processes across various organisms, regulating gene expression through diverse mechanisms at the chromatin, transcriptional, posttranscriptional, translational, and posttranslational levels [2,3]. In plants, lncRNAs have been shown to regulate key physiological activities, such as flowering time [4,5], crop yield [6], fruit development [7], photomorphogenesis [8], gene silencing [9], and responses to biotic and abiotic stresses [10,11,12].

Despite these advances, the functional characterization of plant lncRNAs remains relatively limited, with only a few regulatory mechanisms fully elucidated. For example, the lncRNAs *COLDWARP*, *COLDAIR*, and *COOLAIR* mediate vernalization by silencing FLOWERING LOCUS C (*FLC*) [9,13,14]. The long non-coding RNA LRK Antisense Intergenic RNA (LAIR) interacts with *OsMOF* and *OsWDR5* to promote the expression of leucine-rich repeat receptor kinase (LRK) gene clusters, significantly increasing rice yield [6]. Importantly, the crucial roles of lncRNAs in plant responses to biotic stress and immunity are gradually coming to light. In tomato, lncRNA16397 induces the expression of *SlGRX*, reducing reactive oxygen species (ROS) accumulation and thereby increasing resistance to *Phytophthora infestans* [11]. In *Arabidopsis*, the lncRNA *ELENA1* interacts with the mediator complex subunit MED19a to regulate *PR1* expression, bolstering immunity against pathogens [10]. In rice, the lncRNA *ALEX1* enhances resistance to bacterial blight by modulating the jasmonic acid (JA) pathway [15], whereas the long non-coding RNA *SABC1* helps balance plant immunity and growth by regulating salicylic acid (SA) synthesis [16]. Moreover, lncRNA23468 has been reported to act as a competing endogenous RNA (ceRNA) that may suppress miR482b accumulation, potentially elevating the expression of NBS-LRR genes and contributing to defense against *P. infestans* in tomato [17]. Another study found that overexpressing specific lncRNAs in rice (such as MOIRA1 and MOIRA2) reduced resistance to rice blast disease but simultaneously increased yield, revealing the important role of lncRNAs in coordinating disease resistance and yield traits [18].

Rice (*Oryza sativa* L.), one of the world’s most important staple crops (particularly in Asia and Africa), faces a major threat from rice blast disease caused by the fungus *M. oryzae*. Globally, rice blast can lead to yield losses up to 30%, posing a severe challenge to food security and agricultural economies, with estimated annual losses of up to 66 billion United States dollars [19]. Hence, understanding the molecular mechanisms of rice blast disease is of paramount importance for global food security [20,21].

Earlier research demonstrated that protein-coding genes play significant roles in the defense response of rice against *M. oryzae* [22,23]. However, the involvement of lncRNAs in this process has not been extensively explored. A recent rice telomere-to-telomere genome annotation revealed an additional 1373 protein-coding genes, indicating that the genome annotation is still incomplete, especially in intergenic regions [24]. Such gaps might contribute to false positives in lncRNA identification and thus hamper downstream functional analyses.

It is worth noting that translatome data, which typically capture ribosome-bound mRNAs using techniques like Ribo-seq or RNC-seq, provide direct evidence for differentiating coding from non-coding transcripts [25,26,27]. Because these techniques detect only mRNAs that are actively being translated by ribosomes, filtering based on translatome data allows one to exclude the majority of genuinely protein-coding transcripts, thereby reducing the risk of misclassifying protein-coding genes as lncRNAs. Incorporating such translatome data substantially improves lncRNA annotation accuracy, offering a clear advantage over methods relying solely on conventional genomic or transcriptomic annotations [28].

To more accurately identify and characterize rice lncRNAs expressed during *M. oryzae* infection, we combined multiple software tools and methods suitable for lncRNA identification in rice and developed a pipeline named RiceLncRNA (https://github.com/njausxl/RiceLncRNA) (accessed on 15 March 2025). This pipeline begins by integrating rice translatome data to construct a novel rice protein-coding gene annotation database (the CodingRNA dataset). By merging this dataset with Michigan State University Rice Genome Annotation Project version 7 (MSU v7) [29], we substantially improved annotation completeness (adding approximately 18.9% more gene loci) and accuracy in intergenic regions. With this optimized annotation, we analyzed strand-specific RNA-seq data from IR25 (a monogenic line harboring the blast resistance gene Pikm) and LTH (a susceptible variety) at 0, 12, and 24 h post-inoculation (hpi), and from NPB (a conventional variety) at 0, 24, 48, and 72 hpi. Through systematic analyses of these data, we identified 9003 high-fidelity lncRNAs, with 605 differentially expressed under inoculation—including 415 that respond directly to stress. Notably, the resistant variety IR25 exhibited 293 specific differentially expressed lncRNAs (DELs), whereas LTH showed only 70, suggesting that lncRNAs are substantially involved in the resistance signaling network. Functional enrichment analysis revealed that the putative target genes of these DELs are significantly enriched in multiple hormones signaling pathways, notably, SA, JA, ET, and IAA. By constructing a ceRNA regulatory network and performing WGCNA, we discovered that several core regulatory lncRNAs (e.g., LncRNA.9497.1 and LncRNA.9562.1) either directly target resistance (R) genes, receptor kinases, and disease-resistance proteins, or indirectly regulate them by competitively binding specific miRNAs. Through these complementary mechanisms, these lncRNAs synergistically modulate hormone signaling pathways to improve resistance. This study not only refines the rice genome annotation and establishes a reliable lncRNA identification pipeline but also, more importantly, provides insights into the potential regulatory network of lncRNAs in rice–*M. oryzae* interactions, providing essential theoretical foundations and potential targets for advancing plant immune mechanism research and implementing novel disease-resistant breeding strategies.

## 2. Results

### 2.1. Analysis of the CodingRNA Dataset Assembly

On the basis of translatome data, we constructed a rice protein-coding gene annotation database, which we termed “CodingRNA”. Compared with the MSU v7 reference genome alone, this translatome-based annotation covered approximately 57% of the genome, and merging both annotations yielded an additional ~18.9% of gene loci (Table S2). As shown in Figure S1, several protein-coding genes were identified within regions previously annotated as intergenic in MSU v7. For example, in the region from 4,643,010 to 4,659,242 on chromosome 4 (Figure S1A), MSU v7 had originally classified the entire stretch as intergenic; however, our detailed analysis of the corresponding translatome data revealed a novel protein-coding gene with clear ribosome footprint signals (labeled “CD48328”). This discovery suggests that the locus is more likely to encode a functional protein rather than a non-coding transcript. In summary, by incorporating such findings into the CodingRNA dataset, we improved the accuracy and completeness of rice genome annotation, reducing the likelihood that protein-coding transcripts are not erroneously classified as lncRNAs.

### 2.2. Integrated Translatome and Transcriptome-Based LncRNA Identification Pipeline and Analysis

To systematically uncover rice lncRNAs, we analyzed strand-specific RNA-seq data from *M. oryzae*-inoculated IR25 (carrying the blast resistance gene Pikm) and LTH (susceptible) at 0, 12, and 24 h post-inoculation (hpi), and NPB (a conventional variety) at 0, 24, 48, and 72 hpi. Overall, 40 RNA-seq libraries that have been validated for strand-specificity were utilized (Table S3), though two (LTH-24 h-2 and LTH-24 h-3) were discarded due to poor quality.

After trimming low-quality reads and adapters, we retained approximately 10,070,404,673 high-quality reads, with an average GC content of 48.89% (Table S4). The mean alignment rate to MSU v7 was 96.89%, with an 86.23% rate of correctly paired reads.

**Figure 1:**
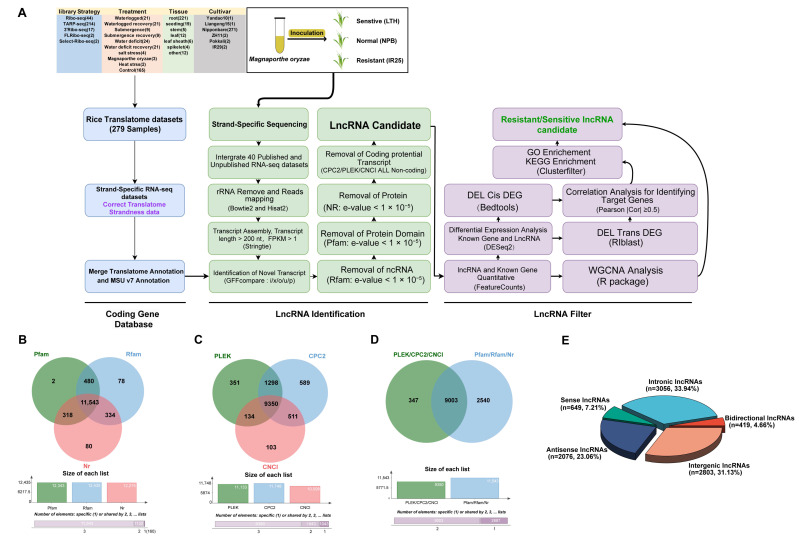
Bioinformatics pipeline for lncRNA identification and visualization of disease-resistance-associated lncRNAs in rice. (**A**) Comprehensive analysis workflow: sequential steps from left to right, illustrating the construction of the “CodingRNA” dataset, criteria and processes for identifying non-coding RNAs (including lncRNAs), and strategies for screening disease-resistance-associated lncRNAs. (**B**–**D**) Venn diagrams: evaluation of coding potential of transcripts using multiple tools and databases, including Pfam, Rfam, Nr, PLEK, CPC2, and CNCI. (**E**) Pie chart: classification of the final identified lncRNAs.

We eventually assembled 114,267 transcripts (≥200 nt, fragments per kilobase of transcript per million mapped reads (FPKM) ≥ 0.5). To mitigate false positives, an updated rice lncRNA identification pipeline (Figure 1A) was implemented. Its key innovation lies in employing the refined CodingRNA dataset to avoid misclassifying protein-coding transcripts as lncRNAs. Through GFFcompare, we extracted transcripts labeled with class codes i, x, o, u, and p (totaling 12,836 candidates; see Figure S2A for an explanation of the class codes). Subsequent filtering against the protein families database (Pfam), RNA families database (Rfam), and NCBI nonredundant protein database (NR) removed additional protein-coding or known non-coding RNAs, giving 11,543 candidates (Figure 1B).

Moreover, we applied the Coding Potential Calculator 2 (CPC2; 11,748 transcripts), Predictor of Long non-coding RNAs and mEssenger RNAs based on an improved K-mer scheme (PLEK; 11,133 transcripts), and Coding-Non-Coding Index (CNCI; 10,098 transcripts) to evaluate the coding potential (Figure 1C). Only transcripts consistently deemed “non-coding” by all three tools were retained (9350). Taking the intersection of these results, we ultimately identified 9003 high-fidelity lncRNAs (Figure 1D; Table S5). Classification indicated 2803 intergenic, 2076 antisense, 419 bidirectional, 3056 intronic, and 649 sense lncRNAs (Figure 1E; Table S6).

A more conventional pipeline that did not integrate translatome data identified 10,464 putative lncRNAs, which included the aforementioned 9003 high-confidence lncRNAs plus an additional 1461 transcripts (Figure S2B). Further analysis revealed that approximately 96.3% of these 1461 transcripts exhibited protein-coding potential, mostly located in insufficiently annotated intergenic regions. When we compared these 1461 transcripts against newly predicted genes in the T2T rice genome (Table S7), about 10.1% (147 transcripts) aligned with those newly predicted coding genes. These findings underscore that incorporating translatome data substantially reduced the false-positive rate in lncRNA identification.

**Figure 2:**
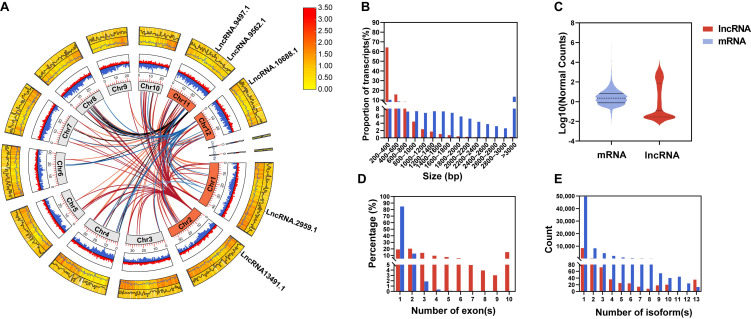
Comparative analysis of the basic characteristics of lncRNAs and mRNAs. (**A**) Circos plot of the trans-putative target genes of four specific lncRNAs. From innermost to outermost: the first ring represents chromosomes, the second ring shows GC content (outer side for lncRNAs, inner side for mRNAs), and the third ring displays heatmaps of gene expression levels and line graphs of gene density (outer side for lncRNAs, inner side for mRNAs). Trans-target gene connections are represented by lines. (**B**) Transcript length distribution. (**C**) Normalized expression level distribution, displayed as violin plots. (**D**) Distribution of the number of exons per transcript. (**E**) Distribution of the number of splice variants per gene locus. Red represents lncRNAs, and blue represents mRNAs.

The final set of 9003 lncRNAs was unevenly distributed across chromosomes (Figure 2A and Figure S3A), with Chr1 bearing the most (1177) and Chr10 the fewest (549). These lncRNAs featured a mean GC content of 41%, notably lower than the ~53% typical of protein-coding genes (Table S8). Approximately 64% spanned 200–400 bp (median 333 nt; Figure 2B), much shorter than mRNAs (median 1008 nt), and displayed lower expression levels (Figure 2C). Notably, ~84.75% contained only one exon (Figure 2D), and they had fewer isoforms than mRNAs (Figure 2E).

Further comparison to publicly available rice lncRNA databases (PlantNATdb, PNRD, RNAcentral, NONCODE, CANTATAdb, and GreeNC) revealed that 3778 (41.96%) of our identified lncRNAs matched known records (E value < 1 × 10^−10^, identity > 80%, coverage > 50%), of which 2446 were shared across all 4 sample sets (Figure S3B).

In summary, the optimized identification pipeline that integrates translatome and transcriptome data not only significantly reduced the false-positive rate and ensured high accuracy of the results, but also provided a convenient workflow and reference standard for future rice lncRNA identification, thereby greatly expanding the rice lncRNA resource.

### 2.3. DELs and Their Differential Expression (DE) Putative Target Genes’ Regulatory Networks Reveal the Mechanisms Underlying Rice Response to M. oryzae Infection

**Figure 3:**
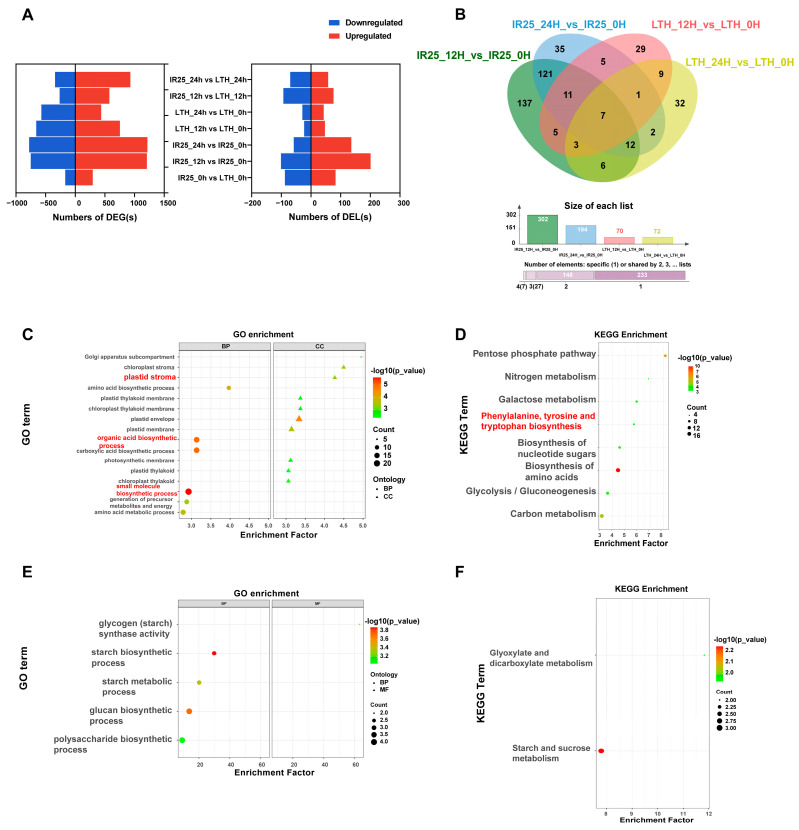
Functional enrichment analysis of DELs and their target DEGs in IR25 and LTH after rice blast infection. (**A**) Bar charts showing the number of DEGs and differentially expressed lncRNAs (DELs), with upregulated genes in red and downregulated genes in blue. (**B**) Venn diagrams of differentially expressed lncRNAs (DELs) between LTH and IR25 before and after inoculation (fold change > 1.5, *p*-value < 0.05). (**C**–**E**) Functional analysis of putative target genes predicted for uniquely expressed DELs in IR25 and LTH, including GO enrichment analysis (**D**,**F**) and KEGG pathway enrichment analysis. The top 20 significant GO terms were selected based on a *p*.adjust < 0.05 cutoff, and the top 10 KEGG pathways were selected with the same significance threshold.

To investigate the transcriptional regulation differences in rice varieties under *M. oryzae* infection and to discover potential resistance- or susceptibility-related lncRNAs, we systematically analyzed the DE of both mRNAs and lncRNAs in the resistant variety IR25 and the susceptible variety LTH at 0 h, 12 h, and 24 h post-inoculation. A comprehensive comparison of transcriptomic differences between inoculated and non-inoculated conditions and across time points revealed 4426 differentially expressed mRNAs (DEGs), including 3821 DEGs and 605 DELs. Of these, 415 DELs and 3338 DEGs were stress-responsive (Table S9; Figure 3A).

When comparing conditions before and after inoculation, the resistant genotype IR25 displayed 293 lineage-specific DELs, whereas the susceptible genotype LTH presented only 70 (Figure 3B). Further analysis indicated that IR25 had the highest number of specific DELs (137) at 12 h post-inoculation, suggesting a faster transcriptional response during the early stage of *M. oryzae* stress. By applying cis- and trans-putative target gene prediction (20 kb upstream and downstream, |r| > 0.5; RIblast combined with correlation analysis), we identified 61 cis- and 341 trans-putative target genes for the 293 IR25-specific DELs (Tables S10 and S11). GO enrichment analysis indicated an association of these putative target genes with processes primarily involved in photosynthesis, “small molecule biosynthetic process” (GO: 0006508) and “organic acid metabolic process” (GO: 0016053), potentially linking them to aromatic amino acid metabolism (Figure 3C). KEGG pathway analysis further revealed significant enrichment in the “phenylalanine, tyrosine, and tryptophan biosynthesis” pathway (osa00400; Figure 3D), with notable enrichment of genes such as LOC_Os01g55870 (chorismate mutase 3, chloroplastic) and LOC_Os09g08130 (indole-3-glycerol phosphate synthase). These genes play critical roles in phenylalanine and tryptophan metabolism, respectively, serving as key precursor nodes for SA and IAA biosynthesis. These results suggest that the DELs specific to IR25 may enhance plant defense against *M. oryzae* by modulating aromatic amino acid metabolic pathways and potentially accelerating the synthesis of resistance-related hormones.

In contrast, the susceptible genotype LTH exhibited only 70 specific DELs, which were associated with 18 cis- and 74 trans-putative target genes (corresponding to 15 and 12 DELs, respectively). GO enrichment showed that these putative target genes were enriched in carbon metabolism-related terms, such as “starch metabolic process” (GO: 0019250) and “sugar metabolic process” (GO: 0006006; Figure 3E). Meanwhile, KEGG analysis indicated that “starch and sucrose metabolism” (osa00500) and “glyoxylate and dicarboxylate metabolism” (osa00630) pathways were significantly enriched (Figure 3F). Thus, under *M. oryzae* stress, LTH-specific DELs appear to be associated with carbon metabolism reprogramming rather than potent defense hormone pathways, suggesting that LTH predominantly undergoes a metabolic adjustment-based stress pattern at early stages, failing to trigger strong hormone-mediated defenses in a timely manner.

Furthermore, we detected 52 DELs shared by IR25 and LTH (Figure 3B), all of which were consistently up- or down-regulated in both varieties. GO enrichment analysis on the cis- and trans-putative target genes of these 52 DELs uncovered terms related to “rhythmic process” (GO: 0048511) and sugar metabolism, such as “hexose metabolic process” (GO: 0019318; Figure S4A). Their KEGG enrichment highlighted the “pentose phosphate pathway” (osa00030), “Calvin cycle carbon fixation” (osa00710), and “amino acid biosynthesis” (osa01230; Figure S4B). These findings point to a conserved role for these commonly responsive genes in energy metabolism and fundamental physiological regulation, helping coordinate basic adaptive responses in both resistant and susceptible genotypes.

We further examined DELs in the NPB variety at 24, 48, and 72 h post-inoculation (Figure S5), observing only a few DELs—just two overlapped with the aforementioned set of 52 DELs—indicating significant differences in how LTH, IR25, and NPB respond at the lncRNA level. This highlights genotype-specific defense mechanisms.

A direct comparison of the susceptible and resistant genotypes (LTH vs. IR25) at 12 h and 24 h post-inoculation revealed 21 lncRNAs co-responsive to *M. oryzae* infection (Figure S4C). Their cis/trans-target enrichment suggested potential synergy between JA and ET biosynthesis, as well as IAA modulation. GO enrichment analysis indicated that phospholipase A1 (*PLA1*) and cysteine synthase genes are closely associated with these 21 lncRNAs, with *PLA1* being crucial for JA biosynthesis during early infection stages and cysteine serving directly as a precursor for ET (Figure S4D) [30,31]. Furthermore, within the KEGG-enriched “plant hormone signal transduction” pathway (Figure S4E), two key genes—*JAZ* (LOC_Os04g32480) and *SAUR* (LOC_Os02g52990)—were identified (Figure S4F,G) [32,33]. The marked upregulation of *JAZ* suggests a potential negative feedback mechanism in JA signaling, whereas the pronounced downregulation of *SAUR* may indicate suppression of IAA, thereby prioritizing the synthesis of defense-related hormones.

Our analysis indicated that the resistant variety IR25 exhibits a more robust transcriptomic response to *M. oryzae* infection than the susceptible LTH, possibly involving lncRNAs related to aromatic amino acid metabolism and hormone signaling. Moreover, we identified a subset of lncRNAs that coordinate JA, ET, and IAA pathways, indicating that JA and ET signals play pivotal roles under *M. oryzae* stress, while IAA signaling functions as an auxiliary route, collectively maintaining a dynamic balance between stress tolerance and immune responses.

### 2.4. Construction of a ceRNA Network Reveals the Role of lncRNAs in Rice Blast Resistance

**Figure 4:**
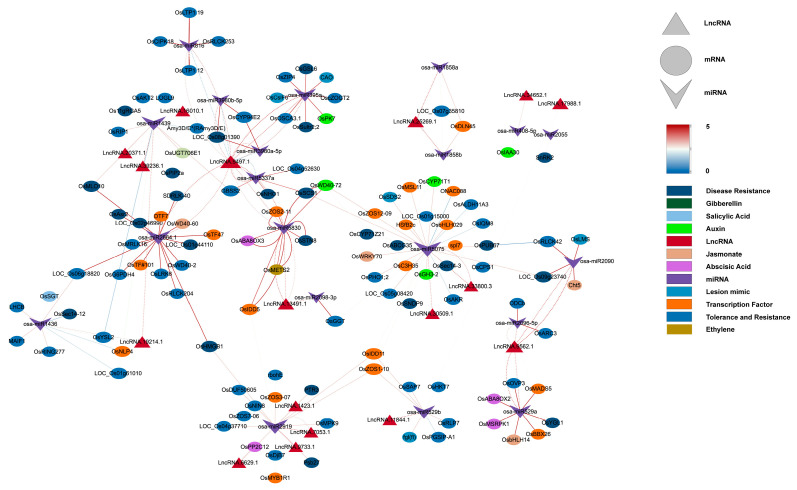
Co-expression ceRNA network of lncRNAs, miRNAs, and mRNAs. Inverted triangles represent miRNAs (purple), triangles represent lncRNAs (red), ovals represent mRNAs, and rectangles represent plant hormones, disease resistance-related genes, and transcription factors (orange). Solid lines indicate connections between miRNAs and mRNAs, while dashed lines represent interactions between miRNAs and lncRNAs. The line color corresponds to the expectation values predicted by psRNAtarget.

This study constructed a ceRNA network containing 20 miRNAs, 17 lncRNAs, and 115 mRNAs (Figure 4), systematically analyzing how lncRNAs indirectly regulate resistance genes and hormone signals by “sponging” miRNAs during *M. oryzae* infection. Four lncRNAs—LncRNA.9497.1, LncRNA.9562.1, LncRNA.13491.1, and LncRNA.33800.3—emerged as major nodes, closely linked to hormone signaling pathways (e.g., JA, IAA, ABA, and gibberellin (GA)), as well as the expression of various resistance-related genes.

LncRNA.9497.1 indirectly regulates several hormone-related genes by “sponging” osa-miR395a, osa-miR2864.1, and osa-miR5830. For instance, osa-miR395a targets *OsSultr2;2* (a sulfate transporter), potentially bolstering sulfur metabolism to supply more substrates for defense [34]. In addition, osa-miR2864.1 targets a variety of receptor kinases, including *OsLRK6* (leucine-rich repeat receptor kinase), *OsMRLK16* (wheat germ agglutinin domain kinase), *OsRLCK204* (receptor-like cytoplasmic kinase), and *SDRLK-40* (receptor-like kinase). Among these, *OsLRK6* is vital for immune signal transduction, whereas *OsMRLK16*, *OsRLCK204*, and *SDRLK-40* facilitate microbe-associated molecular pattern (MAMP) perception and potentially abiotic stress cross-tolerance. Significantly higher expression of these receptor kinases in IR25 at 24 h post-inoculation suggests that certain lncRNAs may facilitate early-stage pathogen recognition through miRNA “sponging”.

Moreover, osa-miR5830 targets *OsABA8ox3* and *OsMETS2*, which regulate ABA degradation and methionine metabolism, respectively. *OsABA8ox3* modulates ABA levels, influencing stress adaptability [35]. In IR25 inoculated with *M. oryzae*, *OsABA8ox3* was notably downregulated by 24 h, whereas *OsMETS2* was upregulated, thus promoting ethylene biosynthesis and reinforcing disease resistance [36]. Thus, in the resistant IR25 line, LncRNA.9497.1 may indirectly be associated with the downregulation of ABA signaling and the upregulation of ethylene signaling via miRNA sponging, potentially contributing to the downstream immune response in rice.

LncRNA.33800.3 indirectly regulates OsWRKY70, *OsCPS1*, and *OsGH3-2* by “sponging” osa-miR5075. OsWRKY70 is a key transcription factor in the JA signaling pathway that positively regulates JA biosynthesis while negatively affecting GA synthesis, thus prioritizing defense over growth [37]. *OsCPS1* participates in GA biosynthesis [38], and *OsGH3-2* is an IAA amino acid synthase implicated in broad-spectrum resistance by limiting auxin levels [39].

In LTH (susceptible) at 12 h post-inoculation, *OsGH3-2* was significantly downregulated, whereas OsWRKY70 was markedly upregulated, indicating that under early pathogen stress, lncRNAs such as LncRNA.33800.3 may simultaneously reduce auxin and raise JA signals to enhance disease resistance.

Furthermore, LncRNA.9562.1 and LncRNA.13491.1 regulate several resistance genes and hormone signals by targeting osa-miR529a, osa-miR5830, and osa-miR2090.

Osa-miR2090 targets *OsRLCK42* (receptor-like protein kinase) and *Cht5* (chitinase). *OsRLCK42* aids in early stress signal transduction, whereas *Cht5* degrades fungal cell walls, a process pivotal to JA-mediated immunity [40]. In LTH, *OsRLCK42* was strongly downregulated, while *Cht5* was upregulated, suggesting partial defense activation yet compromised early signaling in a susceptible background.

Osa-miR529a targets *OsABA8OX2* (ABA metabolic balance) and OsbHLH148 (a transcription factor regulating JA signaling), which contribute to both drought and disease stress responses [41]. In resistant IR25, *OsABA8OX2* tended to be downregulated at 24 h post-infection, while OsbHLH148 was upregulated, reflecting a shift from ABA to JA-driven defenses [42,43].

Osa-miR5830 targets *OsABA8ox3* and interacts with *OsMETS2*, a methionine synthase supporting ethylene biosynthesis [36,44]. This further underscores the intricate hormone crosstalk controlled by hub lncRNAs.

Collectively, these ceRNA interactions suggest that lncRNAs may be involved in hormone pathways (ABA, ET, JA, and IAA) and receptor kinases networks associated with rice blast resistance.

### 2.5. WGCNA Uncovers Key Hormone, R Genes/Proteins, and Receptor Kinase Networks Under M. oryzae Stress

**Figure 5:**
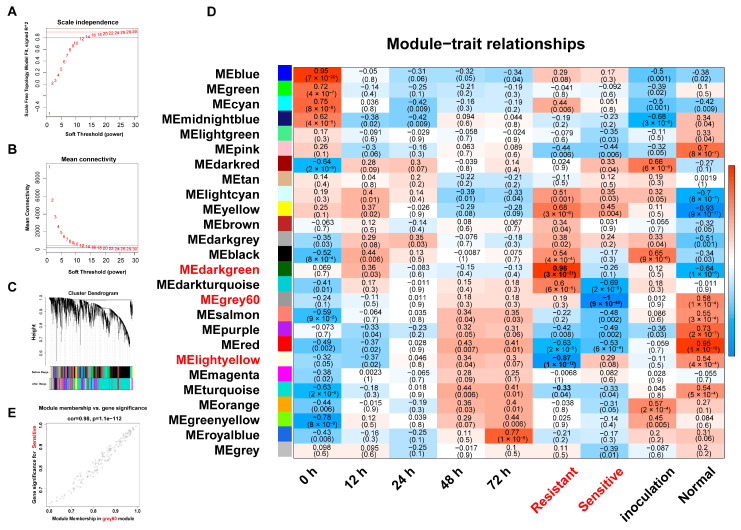
WGCNA of genes and lncRNAs in rice after *M. oryzae* infection. (**A**,**B**) Soft threshold selection for network construction. The optimal soft threshold was chosen based on scale-free topology fit (**A**) and mean connectivity (**B**). (**C**) Hierarchical clustering tree of transcripts in different modules. The dendrogram shows the clustering of transcripts into modules, with each module labeled by a different color. (**D**) Module–trait relationship. The heatmap shows the correlation between module eigengenes and traits. The correlation coefficients and *p*-values indicate the strength and significance of the relationship. (**E**) Scatter plots of transcript significance (TS) versus module membership (MM) for the salt-associated module MEgreys60.

After removing batch effects, normalizing expression, and filtering outliers, a total of 2583 lncRNAs and 15,701 genes were retained for WGCNA (Table S12). With a soft threshold power of 18 (scale-free topology index ~0.85; Figure 5A,B), 26 modules were identified (TOMType = Unsigned, deepSplit = 2, minModuleSize = 30, and mergeCutHeight = 0.2). Notably, around 85.23% of all genes/lncRNAs were grouped into the top 10 modules (Figure 5C).

By correlating module eigengenes (MEs) with phenotypic traits (blast resistance vs. susceptibility; Figure 5D), three notable correlations emerged. The dark-green module showed strong positive correlation with resistance (r = 0.96, *p* = 3 × 10^−21^), while the light-yellow module demonstrated negative correlation with resistance (r = −0.87, *p* = 1 × 10^−12^). The gray60 module exhibited negative correlation with susceptibility (r = −1.0, *p* = 9 × 10^−40^). Scatter plots of transcript significance (TS) vs. module membership (MM; Figure 5E and Figure S6A,B) confirmed these relationships, prompting further network analysis of hub lncRNAs and their putative target genes.

**Figure 6:**
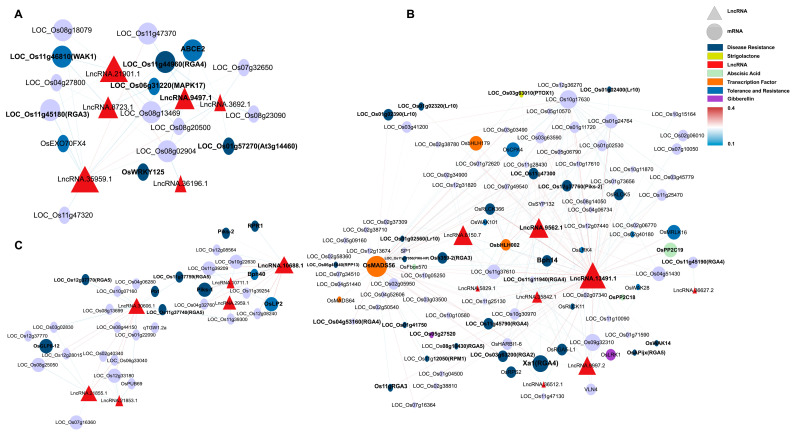
WGCNA network plot showing the correlation between the three modules strongly associated with susceptible and resistant phenotypes. (**A**) The dark-green module is significantly positively correlated with resistance traits (r = 0.96, *p* = 3 × 10^−21^). (**B**) The grey60 module is significantly negatively correlated with susceptibility traits (r = −1.0, *p* = 9 × 10^−40^). (**C**) The light-yellow module is significantly negatively correlated with resistance traits (r = −0.87, *p* = 1 × 10^−12^). The annotations for these related genes are provided in Table S14.

In the dark-green module (Figure 6A; Table S13), LncRNA.9497.1, LncRNA.21901.1, and LncRNA.35959.1 serve as core nodes, associating with disease/stress resistance genes such as wall-associated kinase 1 (*WAK1*), putative disease resistance protein (RGA4), OsWRKY125, and mitogen-activated protein kinase 17 (*MAPK17*). *WAK1* mediates cell wall strengthening and defense signal transduction [45]. Meanwhile, RGA4 and RGA3 are NB-LRR-type resistance genes detecting pathogen effectors [46], and OsWRKY125 is a WRKY TF driving the expression of defense genes [47]. *MAPK17* sits at a crucial position in MAPK signaling cascades under pathogen attack [48].

**Figure 7:**
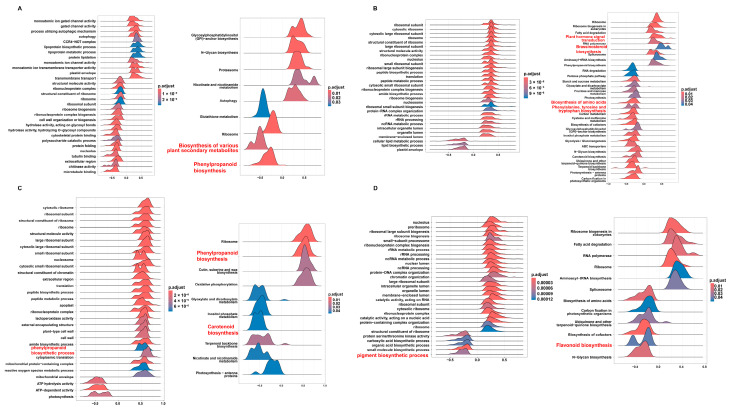
GSEA of gene sets based on four lncRNAs in GO and KEGG pathways: (**A**) lncRNA.9497.1, (**B**) lncRNA.9562.1, (**C**) lncRNA.10688.1, and (**D**) lncRNA.13491.1. The x-axis represents the enrichment score (ES), while the color gradient indicates the adjusted *p*-value (*p*.adjust), reflecting the significance level of the enrichment.

Within the light-yellow module (Figure 7C), LncRNA.10688.1 and LncRNA.36066.1 emerged as central regulatory points. LncRNA.10688.1 is directly linked to RGA5 (LOC_Os11g37740 and LOC_Os12g37770) and *Bph40* (LOC_Os11g39209), both contributing to pathogen-triggered immunity (PTI) and physical defense barriers [49,50]. It also relates to *OsLP2* (LOC_Os12g08240), implying cross-disease and environmental stress crosstalk [51]. In LTH inoculated with *M. oryzae*, RGA5 and *OsLP2* were notably upregulated, whereas *Bph40* was slightly downregulated, suggesting partial immune enhancement yet weaker mechanical defenses in a susceptible background.

*Piks-1* and *Piks-2*, recognized CC–NBS–LRR pairs defending against *M. oryzae* [52], and *Pb1* (LOC_Os04g06280), a durable blast resistance gene from *indica* [53], also converged in this module’s co-expression network, implying multiple layers of early PTI and defense synergy. In terms of stress resistance, *OsPUB69* (LOC_Os12g33180), an E3 ubiquitin ligase gene associated with LncRNA.21855.1, has been shown to play a crucial role in stress tolerance, particularly in protein degradation and cellular homeostasis regulation [54]. Similarly, *OsGLP8-12* (LOC_Os12g28015), associated with LncRNA.36066.1, regulates reactive oxygen species (ROS) levels, which is essential for rice adaptation and disease resistance under oxidative stress conditions [55,56].

The gray60 module features LncRNA.13491.1, LncRNA.9562.1, and LncRNA.9997.2 (Figure 7B), which coordinate multiple receptor kinases (*OsRLCK366* and *OsRLCK5*), resistance genes (RGA5-L1), and hormone regulators (*OsPP2C19*). For example, LncRNA.13491.1 intersects with RGA4, RGA5, *RPM1*, and *Xa1*, all tied to effector-triggered immunity (ETI) [57,58]. It also influences OsMADS56 and OsbHLH179, potentially modulating strigolactone (SL) and GA signals [59,60]. Similarly, LncRNA.9562.1 correlates with *OsRLCK366* (a receptor kinase) and *OsPP2C19* (an ABA regulator) [61,62], facilitating hormone interplay and stress responses [63,64,65]. LncRNA.9997.2 shows correlation with genes involved in GA, ABA, and even cytoskeletal elements via *OsRLCK5* and *VLN4* [66]. Interestingly, based on the expression heatmap of lncRNA within the module (Figure S6C–E), LncRNA.13491.1 and LncRNA.9562.1 peaked in LTH at 12 h but remained low in IR25, indicating potential genotype-specific temporal regulation of immune-associated lncRNAs.

Collectively, these findings suggested that multiple lncRNAs within WGCNA modules coordinate hormone pathways (ABA, GA, and SL), receptor kinases (*OsRLCK5* and *OsRLCK366*), and R genes/proteins (RGA4, RGA5, *Pb1*, *Piks-1*, and *Piks-2*), potentially contributing to both early PTI and downstream ETI, thus supporting a multi-layered defense framework in rice.

### 2.6. Mechanistic Insights into the Functional Roles of Key lncRNAs in Rice Blast Resistance

To examine the mechanistic roles of the top lncRNAs identified by WGCNA, e.g., LncRNA.9497.1, LncRNA.9562.1, LncRNA.10688.1, and LncRNA.13491.1, we performed gene set enrichment analysis (GSEA) for single key lncRNAs, focusing on pathways implicated in secondary metabolism and hormone signals, and structural diagrams for these four lncRNAs were also generated (Figure S7).

LncRNA.9497.1, annotated as an antisense transcript (Figure 7A), was significantly associated with the phenylpropanoid biosynthesis pathway (osa00940) and broader plant secondary metabolite biosynthesis (osa00999; Figure 7A). These metabolic processes contribute to lignin and flavonoid production, which reinforce cell walls and produce antimicrobial compounds. Thus, LncRNA.9497.1 may be linked to disease resistance through these metabolic pathways.

Genes linked to LncRNA.9562.1 were mainly enriched in plant hormone signal transduction (osa04075; Figure 7B and Figure S8), encompassing SA, ET, brassinosteroid (BR), GA, and JA pathways. Critical components, like *NPR1* and *PR1* and ET-related genes, appear among its targets, indicating a capacity to orchestrate hormone crosstalk and secondary metabolism, thereby enabling a rapid immune response to *M. oryzae*.

LncRNA.10688.1 was significantly enriched in the phenylpropanoid biosynthesis pathway (osa00940) and the carotenoid biosynthesis pathway (osa00906; Figure 7C). Phenylpropanoids foster structural defenses and are precursors to SA, while carotenoids enhance antioxidant capacity and can feed into ABA production, thus regulating plant stress adaptation.

LncRNA.13491.1 exhibited notable enrichment in pigment biosynthetic processes (GO: 0046148) and flavonoid biosynthesis (osa00941; Figure 7D). Flavonoids help mitigate oxidative stress and may act as signaling molecules during pathogen attacks, suggesting that LncRNA.13491.1 aligns plant defense responses with redox equilibrium.

Altogether, these key lncRNAs are associated with plant adaptation and defense under *M. oryzae* infection, potentially influencing basic metabolic, secondary metabolic, and multiple hormone signaling pathways, particularly the phenylpropanoid, flavonoid, JA, SA, ET, and IAA pathways. Their multifaceted roles highlight potential targets for breeding disease-resistant rice varieties using gene editing or advanced selection techniques.

### 2.7. qRT-PCR Analysis and LncRNA Cloning Validation

**Figure 8:**
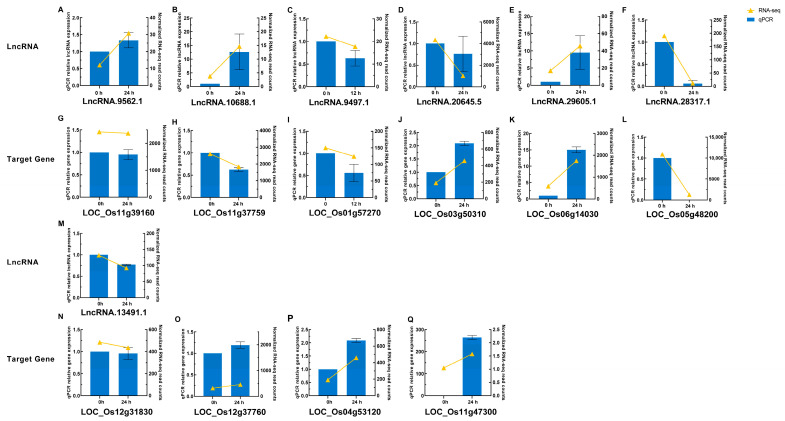
Verification of RNA-seq data by qRT-PCR for DELs and their putative target genes responsive to *M. oryzae*. (**A**–**F**) Expression levels of lncRNAs LncRNA.9562.1 (**A**), LncRNA.10688.1 (**B**), LncRNA.9497.1 (**C**), LncRNA.20645.5 (**D**), LncRNA.29605.1 (**E**), and LncRNA.28317.1 (**F**) at 0 and 24 h post-inoculation (hpi), except for (**C**) which shows 0 and 12 hpi. (**G**–**L**) Expression levels of the corresponding target genes LOC_Os11g39160 (**G**), LOC_Os11g37759 (**H**), LOC_Os01g57270 (**I**), LOC_Os03g50310 (**J**), LOC_Os06g14030 (**K**), and LOC_Os05g48200 (**L**) at the same time points as their respective lncRNAs in (**A**–**F**). (**M**–**Q**) Expression levels of lncRNA LncRNA.13491.1 (**M**) and its corresponding target genes LOC_Os12g31830 (**N**), LOC_Os12g37760 (**O**), LOC_Os04g53120 (**P**), and LOC_Os11g47300 (**Q**) at 0 and 24 hpi. The yellow line with solid triangles represents RNA-seq results, and the blue solid squares represents qRT-PCR results. Error bars indicate the standard error of three replicates.

To validate these bioinformatic results (Table S14), four important lncRNAs and seven of their putative target genes were chosen from key WGCNA modules for quantitative real-time PCR (qRT-PCR; Figure 8A–C,G–I,M–Q). The expression patterns largely matched the RNA-seq data, reinforcing the reliability of differential expression analysis. Another three random DELs and their three putative target genes were likewise confirmed (Figure 8D–F,J–L), supporting the computational pipeline’s accuracy.

One high-priority lncRNA (lncRNA.9562.1) was subsequently cloned, and Sanger sequencing verified its length and sequence fidelity (Figure S9A,B). The cloned sequence fully matched the predictions, indicating the pipeline’s robust annotation. These validation results pave the way for potential functional analyses, such as overexpression or knockout experiments, to assess the phenotypic impacts of this lncRNA on rice disease resistance.

## 3. Discussion

### 3.1. Optimized lncRNA Identification Pipeline and Its Contribution to Rice Genome Annotation

In this study, we developed a more comprehensive rice protein-coding gene database (CodingRNA) by merging translatome data with existing genome annotations, thereby refining the lncRNA identification pipeline (Figure 1A). Compared with conventional annotation approaches, our pipeline demonstrated an enhanced ability to exclude transcripts with protein-coding potential, effectively reducing false-positive lncRNA calls. This improvement aligns with recent telomere-to-telomere genome assembly work [24] reporting the discovery of 1571 additional coding genes beyond MSU v7, underscoring the importance of updated annotation strategies (Table S7).

We identified 9003 high-fidelity lncRNAs (Figure 1D, Table S5), exceeding previous tallies by Wang et al. (4787) and Priyanka et al. (5337) [67,68]. Such discrepancies likely stem from differences in rice varieties, stress conditions, sequencing strategies, and lncRNA pipelines. Notably, 40% of our lncRNAs matched those in public databases (Figure S3B), whereas 60% appeared novel, highlighting possible genotype-, tissue-, or stress-specific expression. Our pipeline incorporates translatome data integration for excluding potential coding transcripts, three-pronged coding potential prediction (CPC2, PLEK, and CNCI) supplemented by Pfam/Rfam/NR filtering, rice-specific TAD features for lncRNA target gene identification, and RIblast for trans-target detection, replacing older methods.

Despite these substantial advances, this study has limitations that warrant further investigation. While we integrated translatome data, employed a three-pronged approach to coding potential prediction, and utilized multiple database filters to improve accuracy, several challenges remain. First, this study focused on specific rice genotypes and specific time points; therefore, it remains unclear whether these resistance-associated lncRNAs maintain consistent expression and regulatory patterns in other *M. oryzae* species or under different environmental conditions. Second, although qRT-PCR validated the expression changes (Figure 8) and cloning confirmed the sequence authenticity of candidate lncRNAs, these results did not directly demonstrate a functional role for these lncRNAs in disease resistance. qRT-PCR confirmed alterations at the transcript level but did not establish a causal relationship between these changes and the observed resistance phenotypes. While this study proposed potential functional mechanisms for lncRNAs based on correlation networks and bioinformatic analyses, these predictions remain tentative without in-depth functional validation. Future research should prioritize functional characterization of candidate lncRNAs using approaches such as CRISPR-Cas9-mediated gene editing to create knockout or overexpression lines. Phenotypic analyses, including disease resistance assays with different *M. oryzae* species, should be performed to confirm the direct impact of these lncRNAs on rice blast resistance. Finally, this work primarily focused on leaf tissue, and future multi-tissue or single-cell omics analyses are needed to reveal the dynamic spatiotemporal expression patterns of lncRNAs.

Nevertheless, by providing an integrated pipeline on GitHub (https://github.com/njausxl/RiceLncRNA) (accessed on 15 March 2025), this study established a solid foundation for more accurate lncRNA discovery and functional studies in rice. The proposed identification pipeline not only effectively reduced false positives but also aligned with the latest telomere-to-telomere genome assembly results, providing new insights for improving rice genome annotation, elucidating resistance mechanisms, and guiding molecular breeding.

### 3.2. LncRNAs Participate in Rice Blast Resistance by Regulating JA, ET, and IAA Signaling Pathways in a Coordinated Manner

We observed that during *M. oryzae* infection, the putative target genes of DELs—which are themselves DEGs—in IR25 (resistant) and LTH (susceptible) varieties were significantly enriched in the JA and ET signaling pathways. This aligned with prior evidence highlighting the importance of the JA pathway in rice blast resistance [67]. Yet, our study also unveiled the ET pathway’s role. For example, some lncRNA targets in both IR25 and LTH—such as *PLA1* (involved in JA biosynthesis) and PCO (affecting ethylene-related transcription factors [30,31])—suggested overlapping ET–JA crosstalk (Figure S4D). This observed pattern may reflect known JA–ET cooperation in defense against necrotrophic pathogens [69,70].

In IR25-specific responses, many DEL targets clustered within aromatic amino acid metabolism, implying potential enhancement of phenylalanine- and tryptophan-derived SA and IAA. Notably, previous studies have demonstrated that IAA and SA exhibit antagonistic effects, and this balance helps conserve energy during pathogen infection, thereby optimizing plant defense responses, which is consistent with our findings [71]. Meanwhile, *JAZ* genes (negative regulators of the JA pathway) [32] and *SAUR* genes (auxin-responsive) [33] also appear among the common DEL targets (Figure S4E–G), implying dynamic hormone crosstalk. Overall, IR25’s greater number of specific DELs (293) versus LTH’s 70 indicates a more robust or earlier activation of these hormone-regulated defenses in the resistant genotype. Conversely, LTH-specific DELs were concentrated in carbon metabolism, illustrating a metabolic shift that might be less effective at early defense induction.

Thus, multiple hormone signals—SA, ET, IAA, and JA—likely appear to be involved in *M. oryzae* stress, and each genotype’s relationship with these pathways may influence the strength and timeliness of immune responses.

### 3.3. LncRNAs Mediate Immune Responses by Regulating RLKs and R Genes/Proteins and by Participating in ceRNA Networks

Our results further indicated that, within the WGCNA modules correlated to blast resistance, RLKs and resistance proteins were markedly enriched. For instance, in the dark-green module, LncRNA.13491.1 interacted with *OsRLCK366* (a receptor-like kinase), OsRGA5-L1 (a resistance protein), and *OsPP2C19* (a signaling regulator). This observation is consistent with findings in mulberry [72], where the lncRNA MuLRR-RLK-AS negatively modulates RLK expression to influence disease resistance.

Moreover, our ceRNA network showed that several lncRNAs—LncRNA.9497.1, LncRNA.9562.1, LncRNA.13491.1, and LncRNA.33800.3—competitively bind specific miRNAs (osa-miR395a, osa-miR2864.1, and osa-miR5830), thereby influencing the expression of *OsSultr2*, OsWRKY70, and *OsGH3-2*, genes affecting sulfur metabolism, JA signaling, and auxin homeostasis, respectively. These discoveries validate the endogenous target mimic (eTM) hypothesis by Franco-Zorrilla et al. (2007) [73]. Similarly, studies in tomato [74] and sweet *sorghum* [75] suggested that lncRNAs strengthen resistance by regulating transcription factors and stress response genes via ceRNA-based interactions.

Furthermore, subcellular localization predictions (Table S15) provided further insights into how these lncRNAs may exert their regulatory functions. For instance, LncRNA.9562.1 exhibited high-confidence localization in extracellular, mitochondrial, and nuclear compartments, suggesting potential roles related to pathogen signal response or nuclear transcriptional events. Conversely, LncRNA.9497.1 is predicted to reside largely in the extracellular region, supporting a putative role in intercellular communication or apoplastic signaling. Such compartment-specific predictions enrich our understanding of how each lncRNA may spatially coordinate defense responses under *M. oryzae* infection.

In summary, beyond their direct modulation of RLKs and R genes/proteins, lncRNAs in rice appear to be involved in indirectly regulating hormone signaling pathways, potentially through ceRNA networks. This suggests a role for lncRNAs in the extensive regulatory network that influences the eventual disease resistance phenotype.

## 4. Materials and Methods

### 4.1. LncRNA Strand-Specific Library Data Sources

A total of 513 translatome datasets were collected from various tissues of *japonica* rice under different experimental conditions [76,77,78,79,80,81], along with 40 strand-specific RNA-seq datasets derived from leaves of multiple *japonica* rice varieties [67,82] (Tables S1 and S3). These datasets served two major purposes: (i) constructing the rice-coding gene dataset for improved annotation and subsequent lncRNA identification, and (ii) conducting differential expression analyses as well as co-expression network inference. Specifically, all 40 strand-specific datasets were used for lncRNA identification and WGCNA, whereas 16 of these datasets were devoted to differential expression analysis. We focused on the monogenic line IR25, harboring the blast resistance gene Pikm, and the susceptible line LTH (Lijiangxintuanhe) [83]. Among the 513 translatome datasets, various experimental replicates spanned stress conditions, such as drought, temperature fluctuation, salinity, submergence, and heavy metals, in addition to multiple tissues (roots, stems, leaves, buds, panicles, and flowers). Detailed information on library construction is provided in the Supplementary Materials.

### 4.2. LncRNA Identification and Classification

A total of 40 strand-specific RNA-seq datasets were used. The downloaded sequence files were converted to FASTQ format using fastq-dump (v2.10.0). Quality control was performed with FastQC (v0.11.9) [84], and summary reports were aggregated using MultiQC (v1.27.1) [85]. Adapter trimming and low-quality read removal were performed using Fastp (v0.20.1) and Trim Galore (v0.6.7) [86,87], applying a Phred quality threshold of 30. Reads with a Phred score below 30 were removed. To eliminate potential rRNA contamination, reads were aligned against an *Oryza sativa* rRNA reference index using Bowtie2 (v2.4.2) [88]. Only unaligned reads were retained for downstream analyses.

The cleaned reads were then aligned to the MSU v7 reference genome using HISAT2 (v2.2.1) [29,89], with the appropriate strand-specific parameters (--rna-strandness FR or --rna-strandness RF). The resulting alignments were converted to BAM format, sorted, and indexed using SAMtools (v1.15) [90]. Mapping statistics were evaluated using RSeQC (v5.0.1) [91] to confirm strand specificity. Libraries with a ‘1++,1−−,2+−,2−+’ value > 0.9 were classified as forward strand-specific (fr-secondstrand), while those with a ‘1+−,1−+,2++,2−−’ value > 0.9 were designated as reverse strand-specific (fr-firststrand). Datasets with values between 0.4 and 0.6 for both categories were deemed non-strand-specific and excluded.

Transcriptome assembly was performed using StringTie (v2.1.5) [92], specifying the correct strand-specific parameters (--fr or --rf). The assembled GTF files from all samples were merged using StringTie merge, and only transcripts longer than 200 nucleotides (nt) with an FPKM (fragments per kilobase of transcript per million mapped reads) ≥ 0.5 were retained.

To identify candidate lncRNAs, the assembled transcripts were compared against the CodingRNA dataset, an integrated rice-coding gene annotation, using GFFcompare (v0.9.8) [93]. Transcripts classified with class codes ‘i’, ‘x’, ‘o’, ‘u’, and ‘p’ were retained. Homology searches were conducted against multiple databases to further refine these candidates. Pfam (v37.0) [94] was used to scan for protein domain signatures (E-value < 1 × 10^−5^). DIAMOND BlastX queries against the NCBI NR database were executed with an 80% identity threshold, a 50% coverage requirement, and an E-value < 1 × 10^−5^ [95]. To eliminate small RNA contaminants, Rfam (v14.10) [96] was queried via cmScan (E-value < 1 × 10^−5^). Transcripts matching known protein-coding sequences or small RNAs were discarded.

The coding potential of the remaining transcripts was assessed using three computational tools: CPC2 (v1.0.1) [97], PLEK (v1.2) [98], and CNCI (v2) [99]. Transcripts with a CPC2 coding probability < 0.5, a PLEK classification score < 0, and a CNCI classification score < 0 were preliminarily retained. Only transcripts consistently classified as non-coding by all three tools were considered for subsequent analyses.

The final high-confidence lncRNA dataset was defined by the following criteria: non-coding classification by CPC2, PLEK, and CNCI, no matched domains or homologs in Pfam and NR, no known sRNA structures in Rfam, transcript length ≥ 200 nt, FPKM ≥ 0.5, and no annotation as a protein-coding gene or overlap with annotated coding loci in the fused CodingRNA dataset or MSU v7. Transcripts meeting these criteria were designated as high-confidence lncRNAs and were subsequently subjected to functional annotation and, where applicable, experimental validation.

Finally, the lncRNAs were categorized based on their genomic locations using GFFcompare and Bedtools closest (v2.30.0) [100]. Intronic lncRNAs (class ‘i’) resided within introns of protein-coding genes. Sense lncRNAs (class ‘o’) overlapped exons of protein-coding genes on the same strand. Antisense lncRNAs (class ‘x’) overlapped exons on the opposite strand. Intergenic lncRNAs (class ‘u’ or ‘p’) were located in gene-desert regions. Bidirectional lncRNAs were identified using Bedtools closest as those located within 1 kb (kilobases) of a protein-coding gene on the opposite strand.

This rigorous pipeline identified 9003 high-confidence rice lncRNAs, providing a refined and comprehensive annotation compared to previous approaches [101].

### 4.3. Identification of Cis- and Trans-Putative Target Genes and Known lncRNAs

To investigate the potential putative target genes of lncRNAs, we first extracted differentially expressed genes (DEGs) and differentially expressed lncRNAs (DELs) from the expression analyses. We then performed Pearson correlation analysis (using the cor function in R) to evaluate the expression correlation between DELs and DEGs, selecting DEL–DEG pairs with an absolute correlation coefficient |r| > 0.5 and a *p*-value < 0.05.

For the prediction of trans-acting lncRNA putative target genes, we employed RIblast to predict lncRNA–gene interactions (setting the interaction energy < −14 kcal/mol and interaction length ≥ 15 bp). We combined this with the Pearson correlation threshold (|r| > 0.5, *p* < 0.05) to identify putative lncRNA–gene associations [102]. Here, trans-acting lncRNAs refer to those that exert regulatory effects by directly binding to target gene mRNAs or through other indirect mechanisms (such as influencing chromatin structure), and their site of action is not adjacent to their own gene locus.

For the prediction of cis-acting lncRNA putative target genes, considering that the median size of topologically associated domains (TADs) in rice is approximately 35 kb [103], we selected a 20 kb window upstream and downstream of the lncRNA locus as the potential cis-regulatory region. If a gene was located within this range and its expression level showed a significant correlation (|r| > 0.5) with the expression level of the lncRNA, the gene was considered a potential cis-acting target gene of that lncRNA. Here, cis-acting lncRNAs refer to those that primarily regulate the expression of their neighboring genes, usually by affecting local chromatin state or transcription processes.

We then downloaded six rice lncRNA databases—PlantNATdb (v1.4) [104], PNRD (v1.0) [105], RNAcentral (v22) [106], NONCODE (v6.0) [107], CANTATAdb (v2.0) [108], and GreeNC (v2.0) [109]—merging them via CD-hit (v4.8.1) [110]. Finally, we utilized Blastn (v2.9.0) [111] (E-value < 1 × 10^−5^, identity > 95%) to compare our candidate lncRNAs with these known datasets, thus identifying novel lncRNAs versus those present in public repositories.

### 4.4. Differential Expression Analysis and Functional Enrichment

We performed differential expression analysis using the DESeq2 (v1.22.1) [112] package in R. Prior to analysis, batch effects were removed, and each group of lncRNAs and mRNAs was analyzed separately. Thresholds of |log2FC| ≥ 1.5 (for lncRNAs) or |log2FC| ≥ 2 (for mRNAs) and adjusted *p* ≤ 0.05 were used to designate significant differential expression. Principal component analysis (PCA) was then conducted via the prcomp package, with visualization through ggplot2 (v3.5.1) [113] and pheatmap (v1.0.12).

Functional enrichment (gene ontology (GO), Kyoto Encyclopedia of Genes and Genomes (KEGG), and gene set enrichment analysis (GSEA)) was carried out using clusterProfiler (v4.10.1) [114], referencing the org.Osativa.eg.db (v0.01) rice database [115]. We applied a significance cutoff of *p*-value < 0.05 for all enrichment analyses.

### 4.5. Transcription Factor Identification and lncRNAs Localization, Visualized in a Circular Plot

Transcription factors (TFs) are crucial in rice’s response to *M. oryzae* stress [21]. Hence, PlantTFDB (v5.0) [116] was utilized to predict TFs within the DEGs, the ceRNA network, and the WGCNA modules. LncRNA subcellular localization was assessed via RNALocate (v3.0) [117], providing an initial insight into nuclear- vs. cytoplasmic-located lncRNAs. Visualization of circular plots was performed using Tbtools [118].

### 4.6. Weighted Gene Co-Expression Network Analysis (WGCNA)

Weighted gene co-expression network analysis (WGCNA) was undertaken to explore interaction relationships among lncRNAs and mRNAs [119], excluding those already used for differential expression analysis. Following expression normalization (via DESeq2) and batch effect removal, low-expression data and outliers were discarded, retaining genes with higher variance (top 75% by median absolute deviation). A soft threshold power of 18 was used, constructing an adjacency matrix via the adjacency function. A topological overlap measure (TOM) was then calculated to define the similarity matrix of lncRNA–mRNA expression.

Hierarchical clustering was performed, and modules were defined or merged using the dynamic tree cut method (deepSplit = 2, minModuleSize = 30, and mergeCutHeight = 0.25). Each module’s eigengene was correlated with specific phenotypes (e.g., rice blast resistance vs. susceptibility), generating correlation matrices. Modules showing an absolute correlation coefficient |r| ≥ 0.8 and *p* < 0.05 were considered significantly associated with the trait. The core hub genes or lncRNAs within those modules were visualized using Cytoscape (v3.10.2) [120].

### 4.7. Single Key LncRNA Analysis

A normalized, batch-corrected expression dataset of both lncRNAs and mRNAs was used for Spearman correlation analyses. For each chosen lncRNA, all rice genes were sorted by the absolute value of their correlation (descending order), creating an ordered gene list.

Next, gene set enrichment analysis (GSEA) was performed via clusterProfiler (v4.14.4) on this ranked list to determine the biological processes or functional categories most associated with the lncRNA in question [114]. The ggplot, ridgeplot, and gseaplot functions in ggplot2 were utilized to visualize the results from GSEA (v1.68.0) [121].

### 4.8. Competing Endogenous RNA (ceRNA) Network Construction

A total of 713 rice miRNAs were sourced from miRBase, forming the foundation for our ceRNA prediction library. psRNATarget (v2) [122], with parameter settings adapted from Zhang et al. [123], was used to predict lncRNA–miRNA and mRNA–miRNA interactions. The integrated mRNA–miRNA–lncRNA co-expression network was finally visualized in Cytoscape (v3.10.2).

### 4.9. qRT-PCR Method and lncRNA Cloning

To validate the reliability of the sequencing results, we randomly selected three long non-coding RNAs (lncRNAs), four key lncRNAs, and ten of their putative target genes for quantitative real-time polymerase chain reaction (qRT-PCR) analysis. Primers for qRT-PCR were designed using Primer3Plus software (https://www.primer3plus.com) (accessed on 1 March 2025) [124] and verified for specificity using PrimerBlast (v1.0.4) [125]. All primers were synthesized by Genscript Biotech (primer sequences are provided in Table S16).

The qRT-PCR experiments were performed using the Applied Biosystems 7500 Real-Time PCR System, with three biological replicates for each sample. The reaction program was as follows: pre-denaturation at 98 °C for 2 min, followed by 40 cycles of denaturation at 98 °C for 2 s, and annealing and extension at 59 °C for 10 s. A melt curve analysis was performed after each run to confirm the specificity of the amplification products. The 18S rRNA gene was used as the reference gene. The relative expression levels of the putative target genes were calculated using the 2^−ΔΔCt^ method.

Specific primers (Table S16) for amplifying the full-length sequences of target lncRNAs were designed based on the RNA-seq data. Total RNA was reverse transcribed into cDNA using SuperScript III Reverse Transcriptase (Invitrogen, Carlsbad, CA, USA). PCR amplification was performed using PrimeSTAR GXL DNA Polymerase (TaKaRa, San Jose, CA, USA), with the following reaction conditions: 98 °C for 5 min (98 °C for 10 s, 60 °C for 15 s, and 72 °C for 1 min per kb) × 30 cycles, and 72 °C for 10 min.

PCR products were separated by agarose gel electrophoresis, purified, ligated into the pNC-Cam1304-35S vector, and then transformed into DH5α competent cells. Positive clones were confirmed by Sanger sequencing (Tsingke, Beijing, China). Sequencing results were aligned with the reference genome to verify the accuracy of the cloned sequences.

## 5. Conclusions

This study provided significant advancements in understanding the roles of lncRNAs in rice’s defense against *M. oryzae*. By creating an optimized lncRNA identification pipeline—incorporating translatome data and existing annotations—we identified 9003 high-confidence rice lncRNAs with improved accuracy. In-depth analyses of differential expression, WGCNA, and ceRNA network construction revealed their critical involvement in multiple hormone signaling pathways (JA, SA, ET, and IAA), as well as in regulating receptor-like kinases and resistance proteins. Key lncRNAs (such as LncRNA.9497.1 and LncRNA.9562.1) appeared to play important roles in rice adaptability and immune response, though further validation is required

These findings offer a comprehensive framework for delineating lncRNA functions in plant immunity and propose molecular targets for breeding resistant rice varieties. Future efforts should emphasize the functional validation of top-candidate lncRNAs through overexpression or knockout lines, exploring their applicability across diverse genotypes and *M. oryzae* strains.

## Data Availability

The datasets are included within the article and its Supplementary Materials. The raw sequencing data have been deposited in the NCBI Sequence Read Archive under accession numbers PRJNA545418 and PRJNA1058262, and in the BIG Data Center at the Beijing Institute of Genomics under accession number CRA003133. In addition, the RiceLncRNA pipeline developed for this study is publicly available on GitHub (https://github.com/njausxl/RiceLncRNA) (accessed on 15 March 2025). All other data and material analyzed in the current study are included in the manuscript and the Supplementary Materials.

## Supplementary Materials

The following supporting information can be downloaded at: https://www.mdpi.com/article/10.3390/plants14172752/s1, Figure S1: Utilization of the CodingRNA dataset improves the accuracy of lncRNA identification. Figure S2: The impact of the CodingRNA dataset on lncRNA identification and classification code annotation categories. Figure S3: Distribution and comparison of lncRNAs across rice varieties and reference databases. Figure S4: Functional analysis of DE putative target genes predicted for co-expressed DELs in IR25 and LTH in response to rice blast stress. Figure S5: Venn diagram of 52 commonly responding lncRNAs in NPB and LTH/IR25 under rice blast stress. Figure S6: Correlation and expression analysis of key WGCNA modules. Figure S7: Structural analysis of key lncRNAs in rice. Figure S8: GSEA KEGG enrichment pathway of lncRNA.9562.1-related genes in plant hormone signal transduction (osa04075). Figure S9: Full-length amplification and sequencing results of lncRNA.9562.1 cloning. Table S1: Translatome data information. Table S2: Comparison of translatome assembly data and the merged dataset with MSU v7 rice annotation. Table S3: Validation information for strand-specific RNA-seq data. Table S4: Statistical summary of strand-specific transcriptome sequencing data. Table S5: Complete GTF annotation information of long non-coding RNAs (lncRNAs). Table S6: Classification of long non-coding RNAs (lncRNAs). Table S7: Distinguishing false-positive lncRNAs from novel protein-coding genes in the telomere-to-telomere (T2T) genome assembly. Table S8: Comparative statistics of lncRNA and gene features. Table S9: Statistics of differential expression analysis for lncRNAs and mRNAs. Table S10: Statistics of cis-putative target genes for differentially expressed lncRNAs across groups. Table S11: Statistics of trans-putative target genes for differentially expressed lncRNAs across groups. Table S12: Expression statistics of filtered and normalized genes and lncRNAs for WGCNA analysis. Table S13: Functional annotations of genes in grey60, dark-green, and light-yellow modules identified by WGCNA. Table S14: Transcriptome-predicted expression results of experimentally validated lncRNAs and mRNAs. Table S15: Multiple subcellular localization prediction and scoring for four key lncRNAs. Table S16: Primer information for qRT-PCR validation experiments.
