# Supplementary material for: Identification of Rice LncRNAs and Their Roles in the Rice Blast Resistance Network Using Transcriptome and Translatome
Source: Plants (Basel). 2025 Sep 3;14(17):2752. doi: 10.3390/plants14172752 (PMC12430395; doi:10.3390/plants14172752)
Supplement: Supplementary file 1 [file plants-14-02752-s001.zip › Figure S5.pdf]

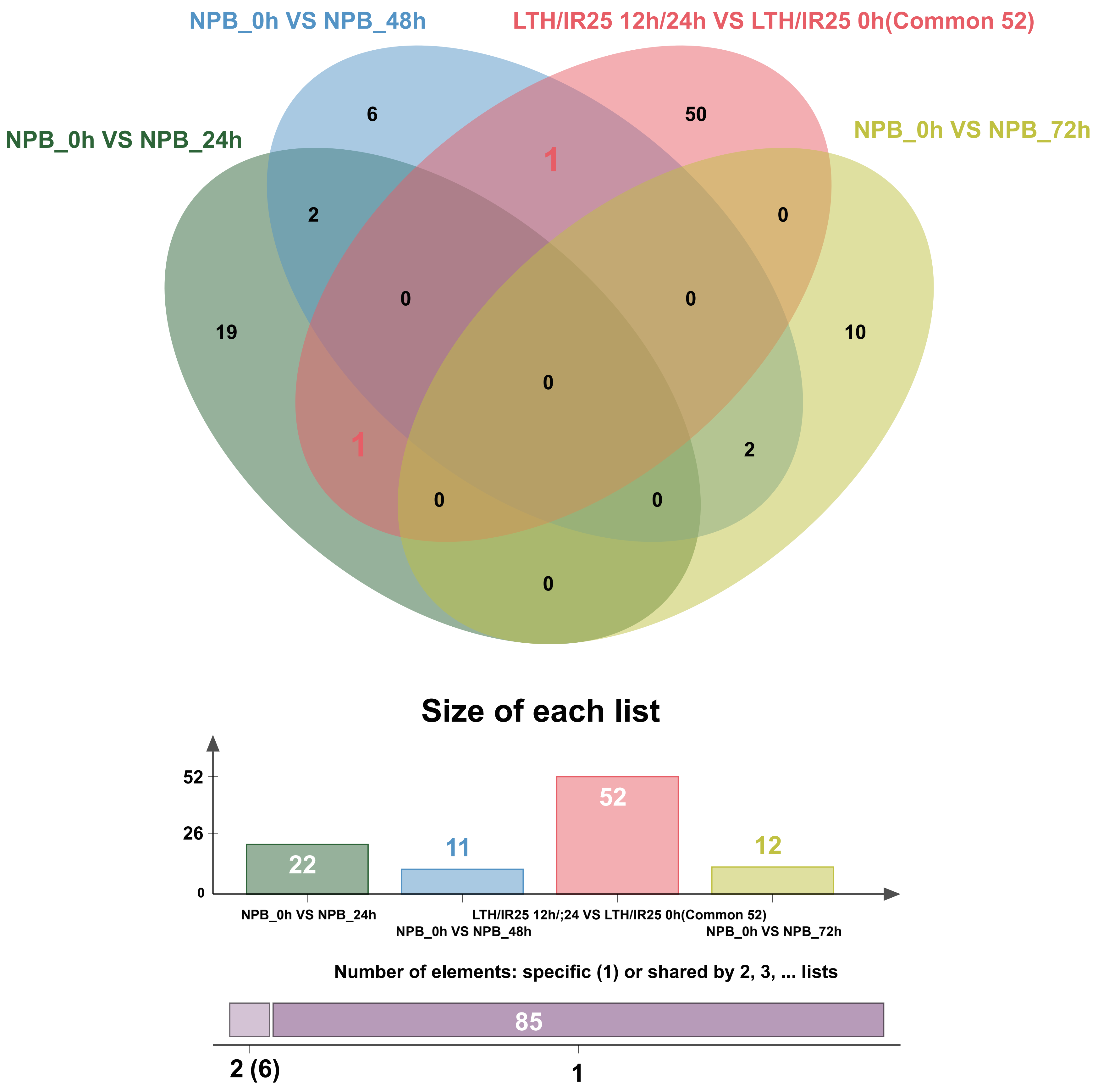

NPB_0h VS NPB_48h
LTH/IR25 12h/24h VS LTH/IR25 0h(Common 52)
NPB_0h VS NPB_24h
NPB_0h VS NPB_72h
6
50
1
2
0
0
0
19
10
0
1
2
0
0
0
Size of each list
52
26
0
22
11
52
12
NPB_0h VS NPB_24h
NPB_0h VS NPB_48h
LTH/IR25 12h/;24 VS LTH/IR25 0h(Common 52)
NPB_0h VS NPB_72h
Number of elements: specific (1) or shared by 2, 3, ... lists
85
2 (6)
1
