# Supplementary material for: Identification of Rice LncRNAs and Their Roles in the Rice Blast Resistance Network Using Transcriptome and Translatome
Source: Plants (Basel). 2025 Sep 3;14(17):2752. doi: 10.3390/plants14172752 (PMC12430395; doi:10.3390/plants14172752)
Supplement: Supplementary file 1 [file plants-14-02752-s001.zip › Figure S8.pdf]

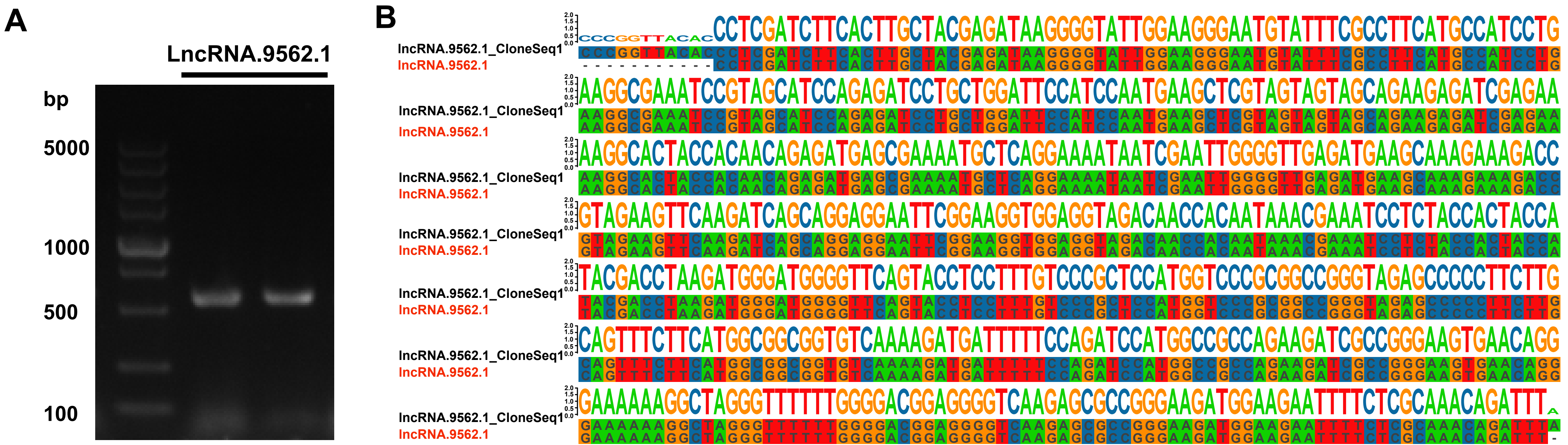

A
LncRNA.9562.1
bp
5000
1000
500
100
B
lncRNA.9562.1_CloneSeq1
lncRNA.9562.1
CCCGGTTACACCCTCGATCTTCACTTGCTACGAGATAAGGGGTATTGGAAGGGAATGTATTTCGCCTTCATGCCATCCTG
AAGGCGAAATCCGTAGCATCCAGAGATCCTGCTGGATTCCATCCAATGAAGCTCGTAGTAGTAGCAGAAGAGATCGAGAA
AAGGCACTACCACAACAGAGATGAGCGAAAATGCTCAGGAAAATAATCGAATTGGGGTTGAGATGAAGCAAAGAAAGACC
GTAGAAGTTCAAGATCAGCAGGAGGAATTCGGAAGGTGGAGGTAGACAACCACAATAAACGAAATCCTCTACCACTACCA
TACGACCTAAGATGGGATGGGGTTCAGTACCTCCTTTGTCCCGCTCCATGGTCCCGCGGCCGGGTAGAGCCCCCTTCTTG
CAGTTTCTTCATGGCGGCGGTGTCAAAAGATGATTTTTCCAGATCCATGGCCGCCAGAAGATCGCCGGGAAGTGAACAGG
GAAAAAAGGCTAGGGTTTTTTGGGGACGGAGGGGTCAAGAGCGCCGGGAAGATGGAAGAATTTTCTCGCAAACAGATTTA
