# Supplementary material for: Identification of Rice LncRNAs and Their Roles in the Rice Blast Resistance Network Using Transcriptome and Translatome
Source: Plants (Basel). 2025 Sep 3;14(17):2752. doi: 10.3390/plants14172752 (PMC12430395; doi:10.3390/plants14172752)
Supplement: Supplementary file 1 [file plants-14-02752-s001.zip › Figure S9.pdf]

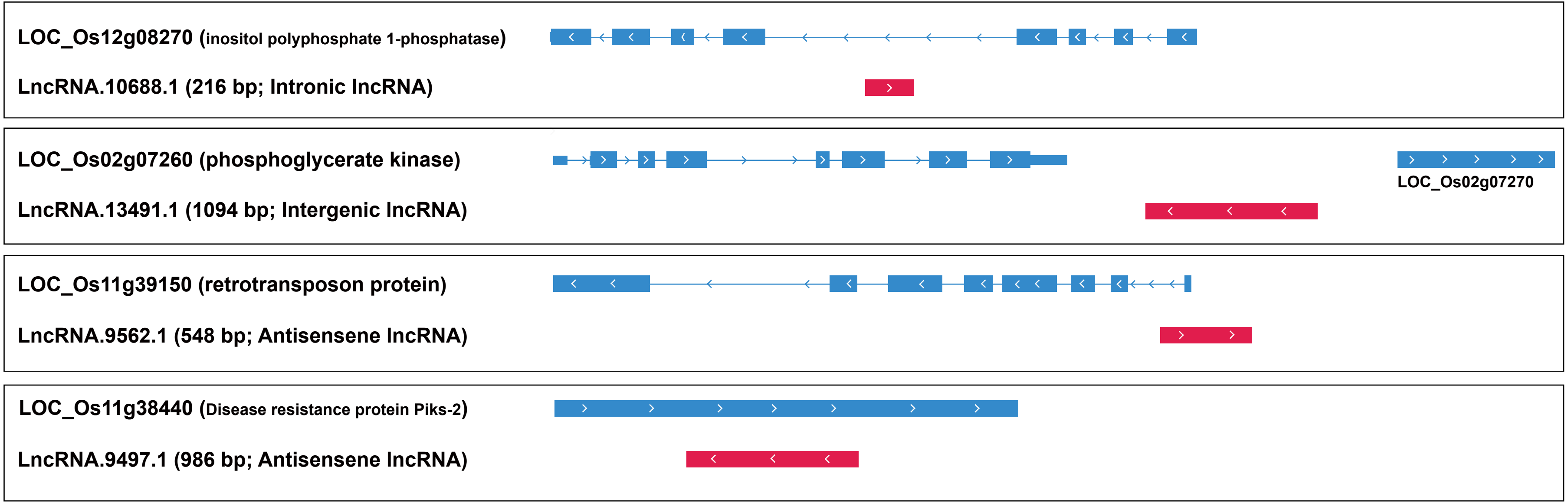

LOC_Os12g08270 (inositol polyphosphate 1-phosphatase)
LncRNA.10688.1 (216 bp; Intronic lncRNA)
LOC_Os02g07260 (phosphoglycerate kinase)
LOC_Os02g07270
LncRNA.13491.1 (1094 bp; Intergenic lncRNA)
LOC_Os11g39150 (retrotransposon protein)
LncRNA.9562.1 (548 bp; Antisensene lncRNA)
LOC_Os11g38440 (Disease resistance protein Piks-2)
LncRNA.9497.1 (986 bp; Antisensene lncRNA)
