# Supplementary material for: Identification of Rice LncRNAs and Their Roles in the Rice Blast Resistance Network Using Transcriptome and Translatome
Source: Plants (Basel). 2025 Sep 3;14(17):2752. doi: 10.3390/plants14172752 (PMC12430395; doi:10.3390/plants14172752)
Supplement: Supplementary file 1 [file plants-14-02752-s001.zip › Supplementary Materials_revised.pdf]

1.Data Sources and Construction of the CodingRNA Database

We searched the NCBI database and online literature repositories using the keywords "Oryza sativa," "rice," "translatome," "Ribo-seq," "Polysome profiling," "RNC-seq," and "3'Ribo-seq." A total of 279 eligible translatome samples were identified[1-6]. Some of these samples included multiple sequencing runs, accumulating to a total of 513 datasets (Table S1), covering various stress conditions such as drought, temperature, salinity, submergence, and heavy metal stress, as well as different rice tissues (roots, stems, leaves, buds, panicles, and flowers).

2. Sequencing Data Sources and Library Preparation Methods

This study utilized strand-specific RNA-seq data from three sources:

2.1 NCBI Sequence Read Archive (SRA) Database (Bioproject: PRJNA545418)

This dataset includes 18 samples derived from the Pikm-resistant rice line IR25 and the susceptible rice line LTH [7]. Detailed information on plant materials and experimental design can be accessed via the GEO database (GSE131641). In brief, rice seedlings were collected at 0, 12, and 24 hours post-inoculation with a mixed strain of Magnaporthe oryzae (rice blast fungus).

To investigate rice responses to pathogen infection, leaf tissues were collected from both rice varieties at 0h (pre-inoculation) and 12h/24h (post-inoculation). Total RNA was extracted using TRIzol reagent (Ambion, USA) and treated with RQ1 DNase (Promega, USA) to remove DNA contamination. RNA quality was assessed using a SmartSpec Plus (Bio-Rad, USA) spectrophotometer (260 nm and 280 nm absorbance) and validated by agarose gel electrophoresis. Ribosomal RNA was removed using a RiboMinus kit (Illumina, USA). The remaining RNA was fragmented at 95°C, followed by end repair and 5' adapter ligation. Reverse transcription was performed using primers containing a 3' adapter sequence and random hexamers. The resulting cDNA was purified and PCR-amplified, and 300–500

bp PCR products were selected, quantified, and stored at -80°C before sequencing. 150 bp paired-end sequencing was performed using the Illumina NextSeq 500 and HiSeq X Ten platforms (ABlife, China).

2.2 BIG Data Center, Beijing Institute of Genomics, Chinese Academy of Sciences (Accession: CRA003133)

This dataset consists of 18 samples from the rice variety Nipponbare (Oryza sativa ssp. japonica). Additional details are available from the BIG Data Center [8]. Leaf tissues were collected at 24, 48, and 72 hours after inoculation with *M. oryzae* strain TMC-1.

Long non-coding RNA (lncRNA) sequencing was performed by NovoGene (Beijing, China). Briefly, total RNA was depleted of ribosomal RNA and used to construct strand-specific RNA libraries with an insert size of 250–300 bp. 150 bp paired-end sequencing was performed on the Illumina HiSeq platform, with each condition having three biological replicates.

2.3 Laboratory-Sequenced Data (PRJNA1058262)

Rice variety Nipponbare was inoculated with *M. oryzae* strains Guy11 and JS153. Leaf samples were collected at 0h (pre-inoculation) and 24h (post-inoculation) for library construction.

Leaf tissues were collected from rice plants inoculated with Guy11, JS153, or 0.4% gelatin, at 0h and 24h. Each sample consisted of pooled leaf tissues from 6–8 plants. Total RNA was extracted using TRIzol reagent (Ambion, USA), and RNA integrity was assessed using an Agilent 2100 Bioanalyzer.

After removing ribosomal RNA, remaining RNA fragments were reduced to 200–300 bp. Reverse transcription was carried out using random primers and reverse transcriptase. During strand-specific library construction, dUTP was used instead of dTTP during the second-strand cDNA synthesis. PCR amplification was performed on 300–400 bp cDNA fragments, followed by quality control using an Agilent 2100 Bioanalyzer. Sequencing was performed on an Illumina high-throughput platform.

All samples were processed and stored according to standard laboratory protocols. These three datasets were chosen to provide a comprehensive analysis of lncRNA diversity and expression patterns across different rice varieties and pathogen strains. Further details on materials and methods are available in the main text.

3. lncRNA Identification Pipeline Code

3.1 CodingRNA Dataset Construction

3.1.1 NCBI Search Expression

(translatome[All Fields] OR Riboseq[All Fields] OR Polysome profiling[All Fields] OR RNCseq[All Fields] OR 3'Ribo*seq[All Fields]) AND "Oryza sativa"[Organism]

Explanation:
This query retrieves translatome-related data (e.g., ribosome profiling, polysome profiling) for *Oryza sativa* from the NCBI database, serving as the foundation for constructing the `CodingRNA` dataset.

3.1.2 Data Download

```
aria2 download_data_url
```

Explanation:
aria2 is used to download raw data files from specified URLs efficiently, allowing batch processing and resumable downloads.

3.1.3 Converting SRR Files to FASTQ

```
fastq-dump --split-3 SRR_input_file -O output_directory
```

Explanation:
fastq-dump (from the SRA Toolkit) converts SRR (Sequence Read Archive) files to FASTQ format and separates paired-end reads. The *--split-3* parameter ensures proper splitting of reads for paired-end data.

3.1.4 Quality Assessment

```
fastqc -t 20 input_file.fastq -o output_directory 2> input_file.log

multiqc output_directory
```

Explanation:
FastQC evaluates read quality, including metrics like per-base quality scores, GC content, and adapter contamination. MultiQC aggregates multiple FastQC reports into one summary, providing an overview for all samples.

3.1.5 Removing Low-Quality Reads and Adapters

3.1.5.1 First Round of Trimming

```
fastp --detect_adapter_for_pe \
--in1 input_1.fastq \
--in2 input_2.fastq \
--out1 ./input_1_fastp.fq \
--out2 ./input_2_fastp.fq \
--json ./input_fastp.json \
--html ./input_fastp.html \
-w 16 \
-q 30 \
-u 20 \
-c \
-n 4 \
2>input_fastp.log
```

Explanation:
Fastp trims adapters and removes low-quality reads (Phred quality score, *-q*, < 30) while handling up to 20% low-quality bases (*-u 20*). The -c parameter corrects mismatched base pairs, ensuring cleaner paired-end data.

3.1.5.2 Second Round of Trimming

```
trim_galore \
--gzip \
-q 20 \
-j 20 \
--length 15 \
--stringency 3 \
-e 0.1 \
--paired \
-o /output_directory \
/input_1_fastp.fq \
/input_2_fastp.fq \
&> input_trimGal.log
```

Explanation:
Trim Galore further refines read quality by trimming remaining adapter

sequences (with a stringency of 3) and discarding reads shorter than 15 bp (--*length 15*). This step helps ensure sufficiently long, high-quality reads.

3.1.6 Contamination Removal (Primarily rRNA)

```
wget -c https://github.com/njausxl/RiceLncRNA/blob/main/s002-OSA_rRNA.fa

bowtie2-build OSA_rRNA.fa OSA_rRNA_index

bowtie2 \
--very-sensitive-local \
--no-unal \
--no-head \
-I 1 -X 1000 -p 28 \
-x OSA_rRNA_index \
-1 input_R1.fastq.gz \
-2 input_R2.fastq.gz \
--un-conc-gz input_clean.fastq.gz \
2> input_MaprRNAStat.xls
```

Explanation:
This step constructs an rRNA index for *Oryza sativa* and removes reads aligning to rRNA. Unaligned reads that pass this filter are retained for downstream analyses.

Selection Reasons:

Bowtie2 is efficient for aligning short reads, and the *--very-sensitive-local* mode is suitable for stricter alignment requirements, enabling efficient removal of contaminating sequences like rRNA. It offers fast alignment speeds.

3.1.7 Alignment and Sorting

```
hisat2-build rice7_all.fa rice7_all_index

hisat2 -p 28 \
-x rice7_all_index \
-1 input_clean.fastq.1.gz \
-2 input_clean.fastq.2.gz \
| samtools view -bS - \
| samtools sort -O bam -@ 28 -o output_sorted.bam - \
2> input.log
```

Explanation:
HISAT2 aligns reads to the reference genome (rice7_all.fa). SAMtools then converts the SAM output to BAM format and sorts the alignments for efficient downstream processing.

3.1.8 Transcript Assembly

```
stringtie -p 28 -v -o output.gtf input_file.bam
```

Explanation:
StringTie assembles transcripts from the BAM file. The *-p* parameter sets the number of threads, while *-v* enables verbose output for monitoring assembly progress.

3.1.9 Merging Transcript Annotations

```
taco_run -p 8 \
-o ./TACO_results \
./gtf.list \
--ref-genome-fasta rice7_all.fa \
--filter-splice-juncs
```

Explanation:
taco_run merges multiple GTF files (listed in gtf.list) into a unified transcriptome annotation. It also applies splice junction filtering to remove low-coverage junctions.

Selection Reasons:

When consolidating a large number of GTF files into a unified transcriptome annotation, TACO was selected in favor of StringTie. TACO offers improved accuracy, particularly in its handling of low-coverage assembled transcripts. By filtering these low-confidence transcripts, TACO enhances the completeness and reliability of the final annotatio

3.1.10 Comparing Translatome Annotation with MSU v7

```
gffcompare \
-r CodingRNA.gtf \
-o MSU_v7_VS_codingRNA \
./MSU_v7.gtf
```

Explanation:
GFFcompare compares the newly assembled annotation (CodingRNA.gtf) against the MSU v7 reference annotation, identifying novel or corrected transcripts that are missing from MSU v7.

3.1.11 Merging Translatome Annotation and MSU_v7 for lncRNA Discovery

```
stringtie --merge -i -g 0 \
MSU_v7.gtf \
CodingRNA.gtf \
-o CodingRNA_MSUv7_merged.gtf
```

Explanation:
StringTie *--merge* consolidates the MSU_v7 annotations with the CodingRNA-derived transcripts. The merged GTF file (CodingRNA_MSUv7_merged.gtf) is then used in lncRNA discovery.

3.2 lncRNA Identification

3.2.1 Data Download

```
wget -c input_data_url
```

Explanation:
RNA-seq data for lncRNA analysis is fetched in a similar manner as for building the CodingRNA dataset. The same quality control steps described in Sections **3.1.2–3.1.6** are applied.

3.2.7 Strand-Specific Alignment

```
hisat2 \
--dta -t \
-p 40 \
--rna-strandness RF \
-x rice7_all_index \
-1 inupt_clean_1.fq.gz \
-2 inupt_clean_2.fq.gz \
-S inupt.sam \
&> inupt_hisat2.log
```

Explanation:
HISAT2 aligns paired-end reads to the genome while preserving strand information (*--rna-strandness RF*, assuming reverse-strand specificity). The *--dta* and *-t* parameters facilitate transcript assembly and timing statistics, respectively.

Selection Reasons:

HISAT2 is fast and accurate for aligning reads to eukaryotic genomes, especially suitable for strand-specific RNA-seq. The *--rna-strandness FR or RF* parameter should be enabled according to the library preparation method.

3.2.8 SAM Conversion and Sorting

3.2.8.1 Converting SAM to BAM

samtools view --threads 40 -S -h inupt.sam -b -o inupt.bam

Explanation:
Converts the SAM file to a compressed, indexable BAM format, essential for efficient downstream operations.

3.2.8.2 Sorting the BAM

samtools sort --threads 40 inupt.bam -o inupt_sorted.bam

Explanation:
Sorting by genomic coordinates is necessary for transcript assembly and coverage calculations.

3.2.9 Verifying Strand-Specific Data

rseqc infer_experiment.py -r rice7_all.fa -i input_sorted.bam

Explanation:

"1++,1--,2+-,2-+" > 0.9 => Forward strand-specific (fr-secondstrand)
"1+-,1-+,2++,2--" > 0.9 => Reverse strand-specific (fr-firststrand)
Approximately 0.4–0.6 for both categories => Non-strand-specific
This step checks alignment statistics using RSeQC to confirm strand specificity. Any dataset not meeting these thresholds is excluded from further processing.

Selection Reasons:

Specifically designed for assessing strand specificity in RNA-seq data, enabling rapid determination of library orientation and evaluating whether sequencing data meet expected strand-specific requirements.

3.2.10 Transcript Assembly and Merging

3.2.10.1 Assembly

```
stringtie --rf input_sorted.bam -o input.gtf -p 40
```

Explanation:
For RF (fr-firststrand) libraries, the *--rf* option ensures transcripts are assembled in the correct orientation.

Selection Reasons:

StringTie can quickly assemble transcripts and allows setting parameters for strand-specific data (*--rf* and *--fr*). It is accurate for multi-exon gene annotation and suitable for large-scale RNA-seq analysis.

3.2.10.2 Merging

```
stringtie --merge \
-i -g 0 \
input_list.gtf \
-l LncRNA \
-o LncRNA_merge.gtf
```

Explanation:
Multiple GTF files (listed in input_list.gtf) are merged, designating transcripts under the label "LncRNA" for subsequent steps.

3.2.11 Extracting Candidate lncRNAs

3.2.11.1 Comparison

```
gffcompare \
-r CodingRNA_MSUv7_merged.gtf \
-o LncRNA_Coding_Compare \
LncRNA_merge.gtf
```

Explanation:
GFFcompare compares the merged transcripts (LncRNA_merge.gtf) against the comprehensive annotation (CodingRNA_MSUv7_merged.gtf). Class codes reveal how each transcript overlaps with known annotations.

3.2.11.2 Extraction

```
awk '$3=="i" || $3=="u" || $3=="x" || $3=="o" || $3=="p" {print "\"" $5 "\""}'
LncRNA_Coding_Compare.gtf.tmap > LncRNA_iuxop.id
```

Explanation:
This command selects only transcripts labeled with class codes 'i', 'u', 'x', 'o', and 'p' (intronic, intergenic, antisense, sense, and potentially processed, respectively).

3.2.11.3 Merging GTF Annotations

```
LC_ALL=C fgrep -f LncRNA_iuxop.id LncRNA_merge.gtf > LncRNA_ixoup.gtf
```

Explanation:
This retains only the previously extracted transcripts, unifying them into a single annotation file (LncRNA_ixoup.gtf).

3.2.11.4 Extracting Candidate lncRNA Sequences

```
gffread LncRNA_ixoup.gtf -g rice7_all.fa -w LncRNA_ixoup.fa
```

Explanation:
Extracts the full nucleotide sequences of the selected transcripts from the reference genome FASTA file (rice7_all.fa), creating LncRNA_ixoup.fa.

3.2.12 Pfam and NR Database Searches to Remove Coding Genes

3.2.12.1 Pfam Scan

```
pfam_scan.pl -cpu 24 -fasta LncRNA_ixoup.fa -dir pfamdata -outfile
LncRNA_ixoup_pfam.out
```

Explanation:
Checks for known protein domains against the Pfam database. Transcripts with hits below an E-value < 1e-5 are flagged as protein-coding and removed.

Selection Reasons:

Utilizes the official protein domain database to rapidly identify and exclude transcripts containing conserved protein domains, thereby reducing false positives in transcript classification.

3.2.12.2 NR Search

```
diamond blastx \
--db nr_diamond \
--query LncRNA_ixoup.fa \
-e 1e-5 --outfmt 6 \
--more-sensitive \
--max-target-seqs 1 \
--threads 24 \
--quiet \
--id 80 \
--subject-cover 50 \
--query-cover 50 \
--out ./LncRNA_ixoup_NR_matches.txt
```

Explanation:
Aligns transcripts to the NCBI non-redundant protein database (NR). Any transcript with an E-value < 1e-5 is considered protein-coding and filtered out.

Selection Reasons:

The most comprehensive non-redundant protein sequence database officially maintained by NCBI, ensuring broad coverage and reliability. When used with DIAMOND, it enables highly efficient and accurate sequence alignment for protein-coding potential analysis.

3.2.13 Rfam Scan to Remove Known Small RNAs

```
cmscan --cpu 24 \
-Z 720 \
--cut_ga --rfam --nohmmonly --fmt 2 \
--tblout LncRNA_ixoup_rfam.tblout \
-o LncRNA_ixoup_Rfam.result \
--clanin Rfam.clanin \
Rfam.cm \
LncRNA_ixoup.fa
```

Explanation:
Searches Rfam for conserved structural RNAs. Transcripts matching known small RNAs (E-value < 1e-5, length >= 200 bp) are excluded. *(Note: Changed ">" to ">=" for consistency.)*

Selection Reasons:

Utilizes the Rfam Covariance Model (CM) to accurately detect small RNAs, including rRNA, tRNA, and snRNA, facilitating the exclusion of non-target transcripts. As an official database, it ensures high sequence credibility and precise alignment results.

3.2.14 Coding Potential Prediction

3.2.14.1 CNCI

```
python2 CNCI.py -f $INPUT_FILE -o $OUTPUT_DIR -m pl -p 10
```

Explanation:
Calculates non-coding scores using CNCI. Transcripts with CNCI scores < 0 are retained, indicating a non-coding prediction.

3.2.14.2 CPC2

```
CPC2.py -i $INPUT_FILE -o $OUTPUT_FILE 2>cpc2.log
```

Explanation:
Evaluates ORF-related features to produce a coding_probability. Transcripts with coding_probability < 0.5 are retained.

3.2.14.3 PLEK

```
PLEK.py -fasta $INPUT_FILE -out $OUTPUT_FILE -thread 10
```

Explanation:
Uses a k-mer approach for coding discrimination. Transcripts with scores < 0 are considered non-coding.

Selection Reasons:

These three tools evaluate transcript coding potential from distinct algorithmic perspectives, and their combined use enhances the reliability of non-coding transcript identification.

CNCI: Includes a plant-specific mode (-p), improving classification accuracy for plant species.

CPC2: An upgraded version of CPC, integrating multiple species, including plants, for improved performance in plant transcriptome analysis.

PLEK: Employs a k-mer-based approach, offering a simple and efficient implementation.

All three tools are well-suited for plant transcriptome analysis, with easy installation, fast execution, and enhanced accuracy when intersecting their results to reduce misclassification.

3.2.15 Intersection of Non-coding Predictions

Rfam ∩ Pfam ∩ NR ∩ PLEK ∩ CPC2 ∩ CNCI

Explanation:
Only transcripts passing all *six* non-coding filters (Rfam, Pfam, NR, CPC2, CNCI, and PLEK) are designated as candidate lncRNAs, ensuring a high-confidence set.

Final Outcome

A total of 9,003 candidate lncRNAs remain following these multi-stage filtering steps, providing a robust dataset for downstream analyses.

More detailed identification and screening process code is hosted on GitHub (https://github.com/njausxl/RiceLncRNA/).

Reference

1. Kajala, K.; Gouran, M.; Shaar-Moshe, L.; Mason, G.A.; Rodriguez-Medina, J.; Kawa, D.; Pauluzzi, G.; Reynoso, M.; Canto-Pastor, A.; Manzano, C.; et al. Innovation, Conservation, and Repurposing of Gene Function in Root Cell Type Development. *Cell* 2021, *184*, 3333-3348.e19, doi:10.1016/j.cell.2021.04.024.
2. Zhu, W.; Xu, J.; Chen, S.; Chen, J.; Liang, Y.; Zhang, C.; Li, Q.; Lai, J.; Li, L. Large-Scale Translatome Profiling Annotates the Functional Genome and Reveals the Key Role of Genic 3' Untranslated Regions in Translatomic Variation in Plants. *Plant Commun* 2021, *2*, 100181, doi:10.1016/j.xplc.2021.100181.
3. Reynoso, M.A.; Borowsky, A.T.; Pauluzzi, G.C.; Yeung, E.; Zhang, J.; Formentin, E.; Velasco, J.; Cabanlit, S.; Duvenjian, C.; Prior, M.J.; et al. Gene Regulatory Networks Shape Developmental Plasticity

of Root Cell Types under Water Extremes in Rice. *Developmental Cell* 2022, *57*, 1177-1192.e6, doi:10.1016/j.devcel.2022.04.013.

4. Yang, X.; Song, B.; Cui, J.; Wang, L.; Wang, S.; Luo, L.; Gao, L.; Mo, B.; Yu, Y.; Liu, L. Comparative Ribosome Profiling Reveals Distinct Translational Landscapes of Salt-Sensitive and -Tolerant Rice. *BMC Genomics* 2021, *22*, 612, doi:10.1186/s12864-021-07922-6.
5. Reynoso, M.A.; Kajala, K.; Bajic, M.; West, D.A.; Pauluzzi, G.; Yao, A.I.; Hatch, K.; Zumstein, K.; Woodhouse, M.; Rodriguez-Medina, J.; et al. Evolutionary Flexibility in Flooding Response Circuitry in Angiosperms. *Science* 2019, *365*, 1291–1295, doi:10.1126/science.aax8862.
6. Xu, Q.; Liu, Q.; Chen, Z.; Yue, Y.; Liu, Y.; Zhao, Y.; Zhou, D.-X. Histone Deacetylases Control Lysine Acetylation of Ribosomal Proteins in Rice. *Nucleic Acids Res* 2021, *49*, 4613–4628, doi:10.1093/nar/gkab244.
7. Fan, J.; Quan, W.; Li, G.-B.; Hu, X.-H.; Wang, Q.; Wang, H.; Li, X.-P.; Luo, X.; Feng, Q.; Hu, Z.-J.; et al. circRNAs Are Involved in the Rice-Magnaporthe Oryzae Interaction. *Plant Physiol.* 2020, *182*, 272–286, doi:10.1104/pp.19.00716.
8. Wang, L.-L.; Jin, J.-J.; Li, L.-H.; Qu, S.-H. Long Non-Coding RNAs Responsive to Blast Fungus Infection in Rice. *Rice* 2020, *13*, 77, doi:10.1186/s12284-020-00437-w.
