# Supplementary figures and images for: Identification of Rice LncRNAs and Their Roles in the Rice Blast Resistance Network Using Transcriptome and Translatome
Source: Plants (Basel). 2025 Sep 3;14(17):2752. doi: 10.3390/plants14172752 (PMC12430395; doi:10.3390/plants14172752)

A

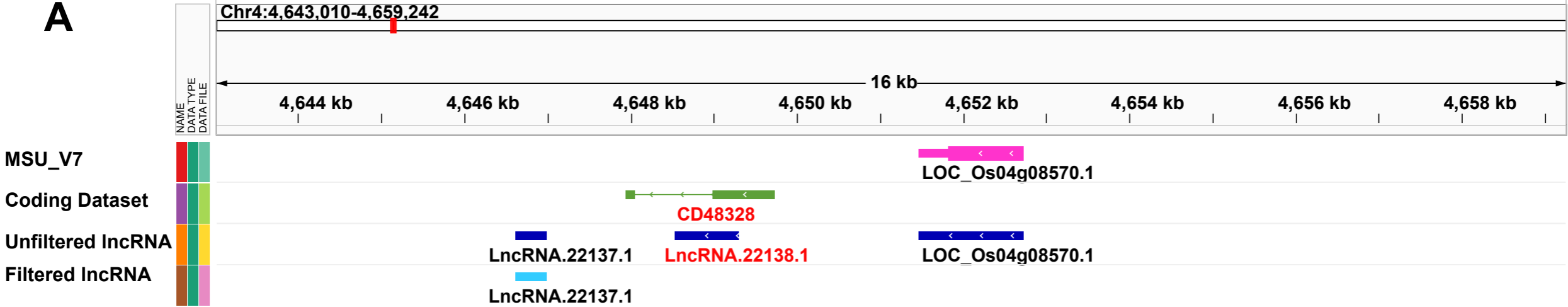

B

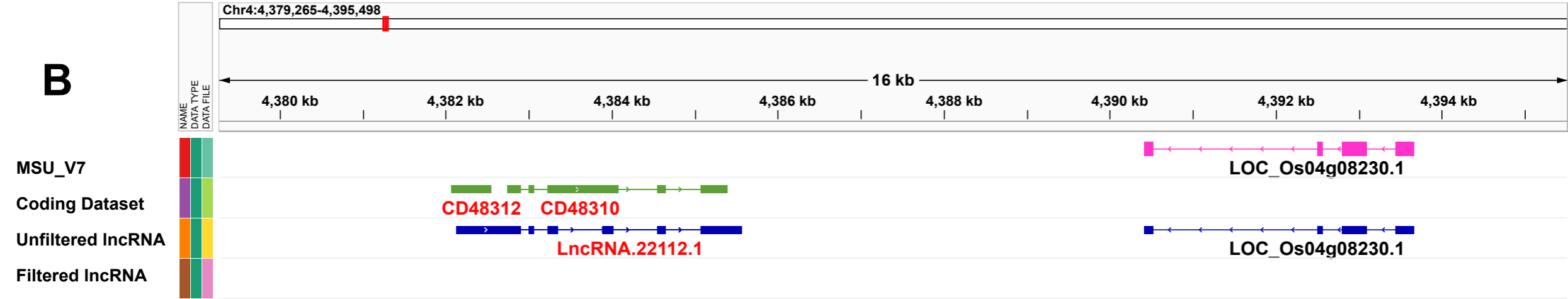

C

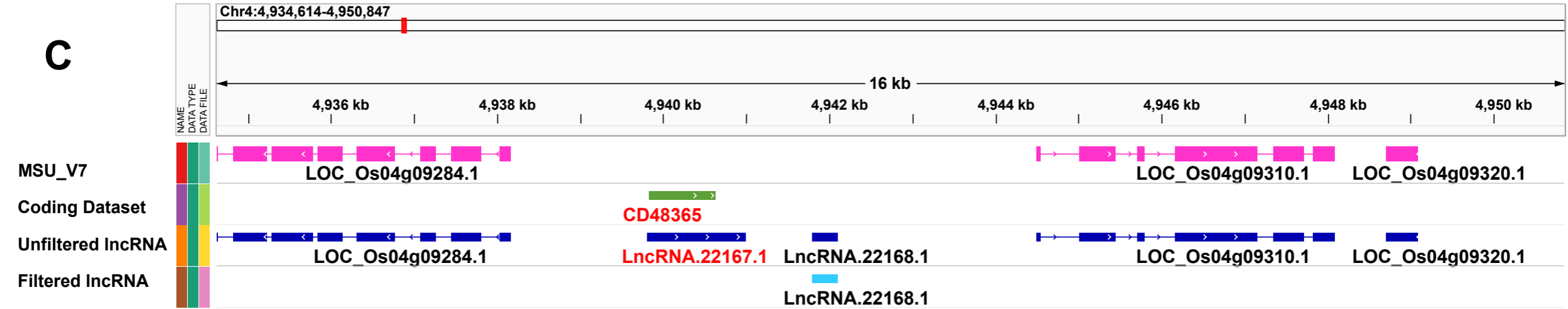

D

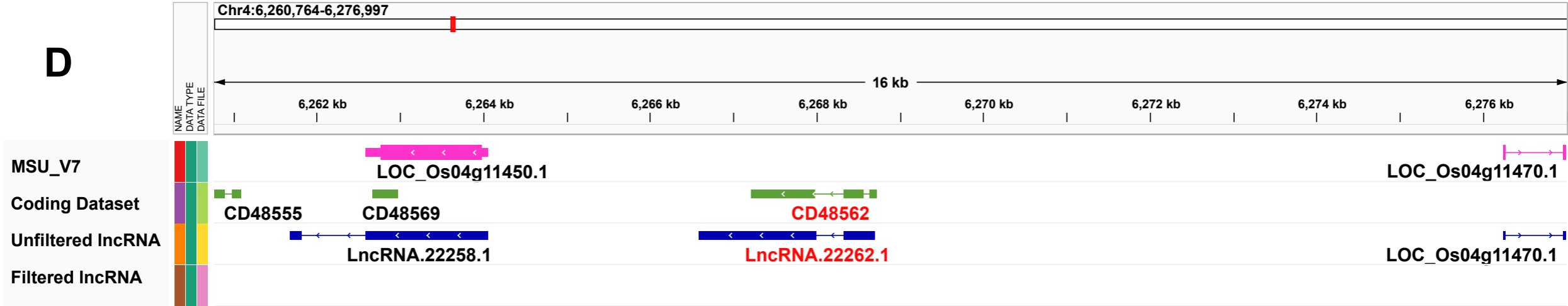

Supplement: Supplementary file 1 [file plants-14-02752-s001.zip › Figure S1.pdf]

A

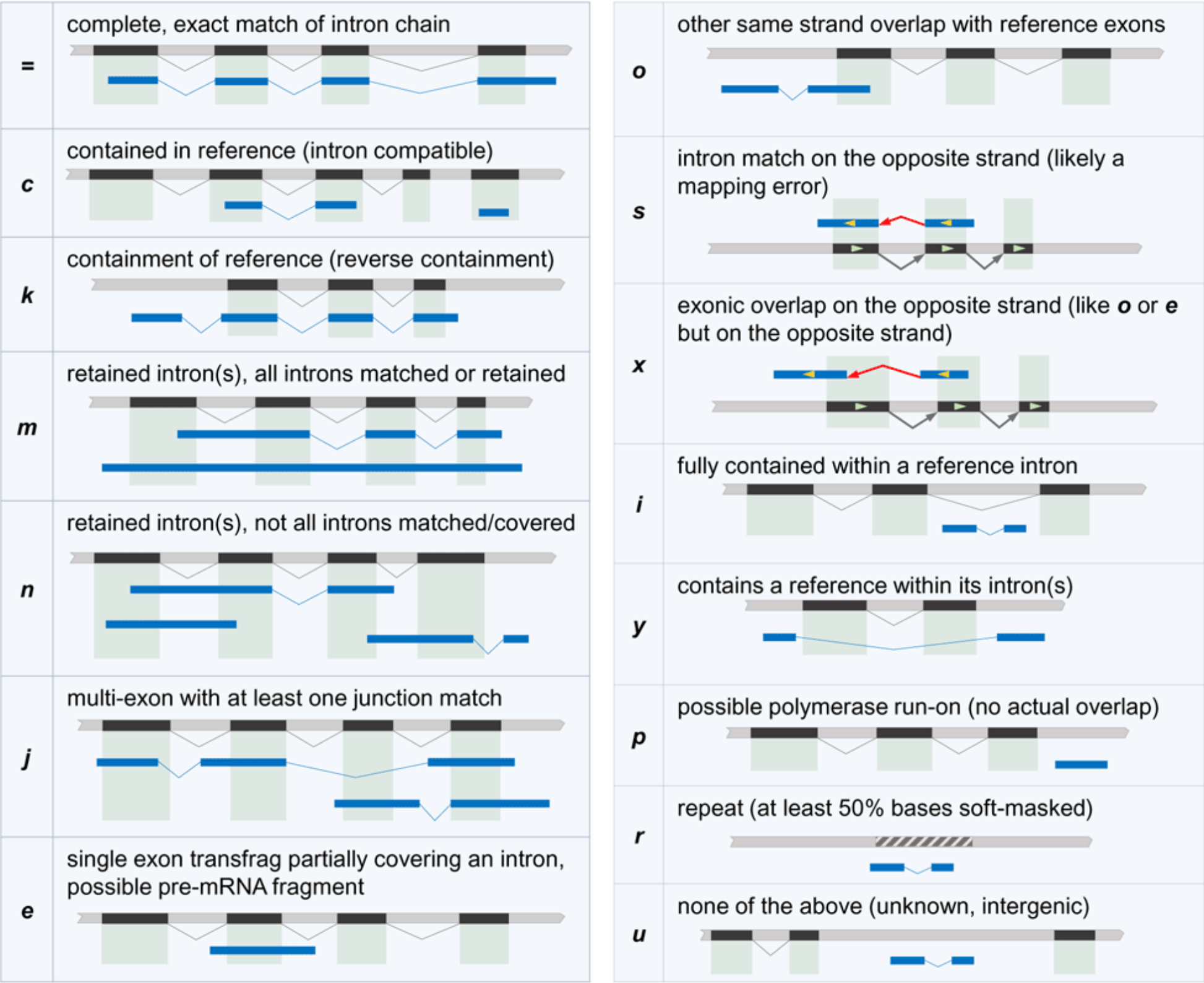

B

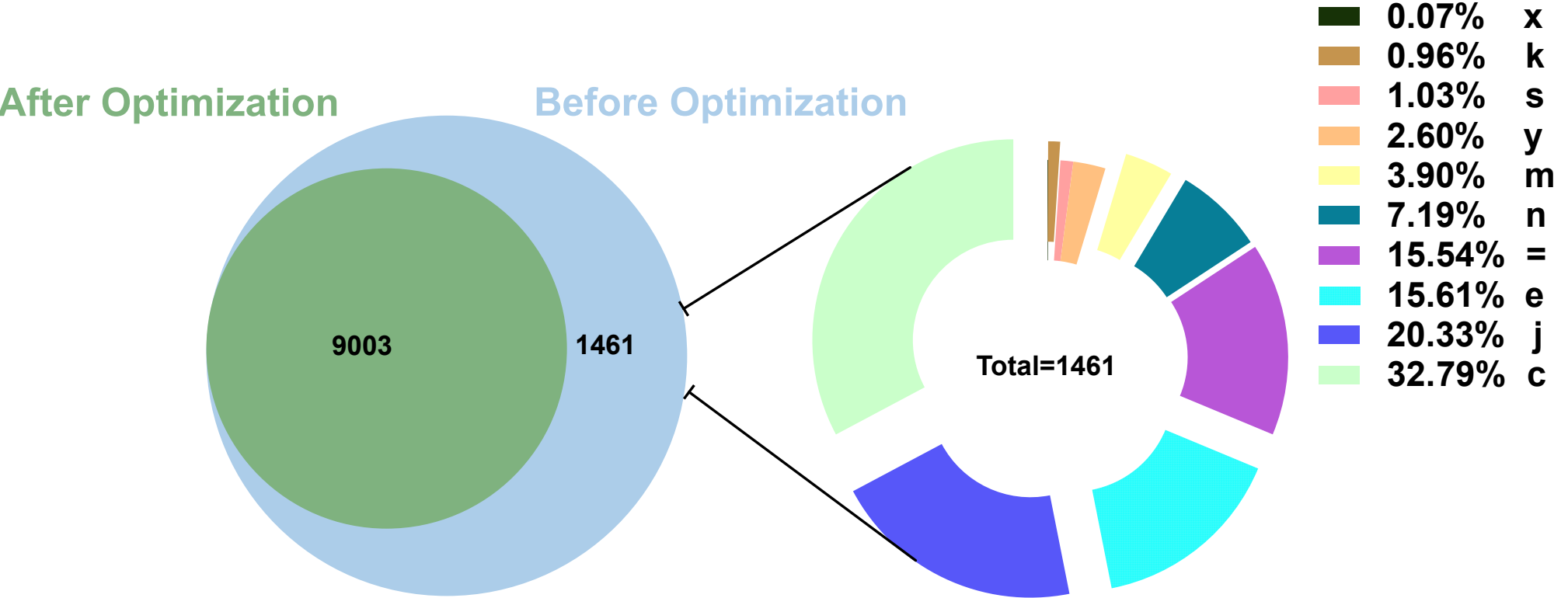

Supplement: Supplementary file 1 [file plants-14-02752-s001.zip › Figure S2.pdf]

**A**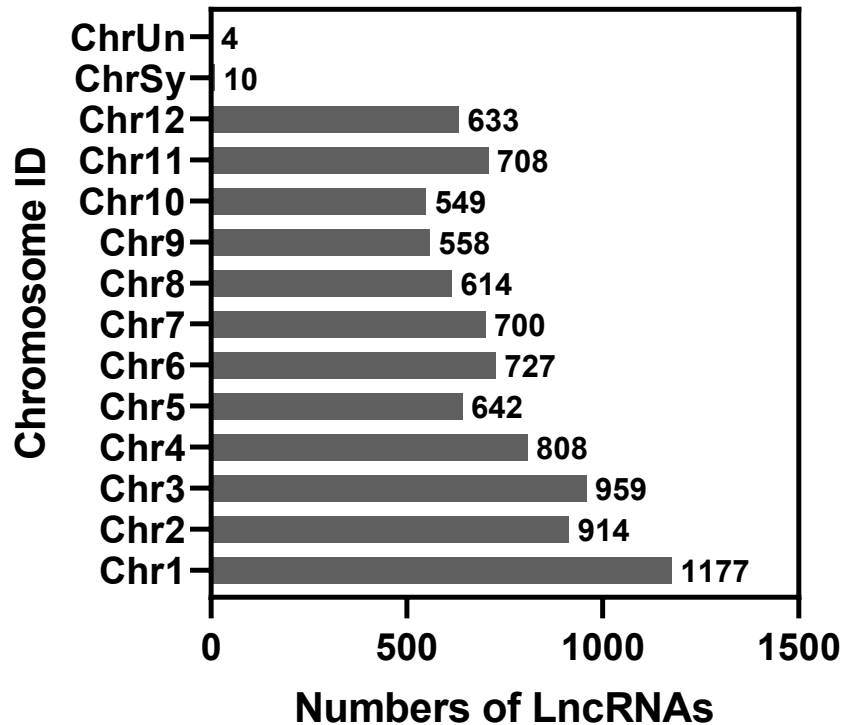**B**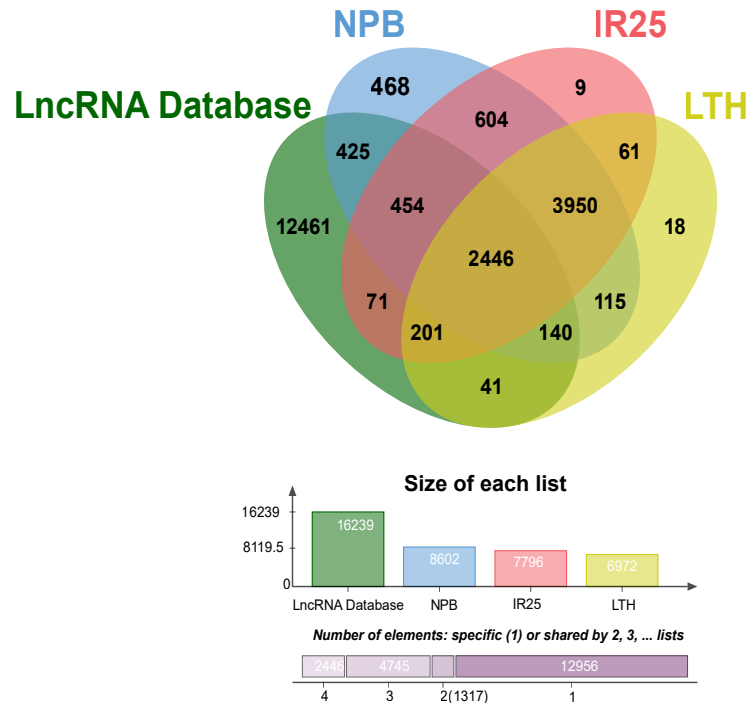

Supplement: Supplementary file 1 [file plants-14-02752-s001.zip › Figure S3.pdf]

A

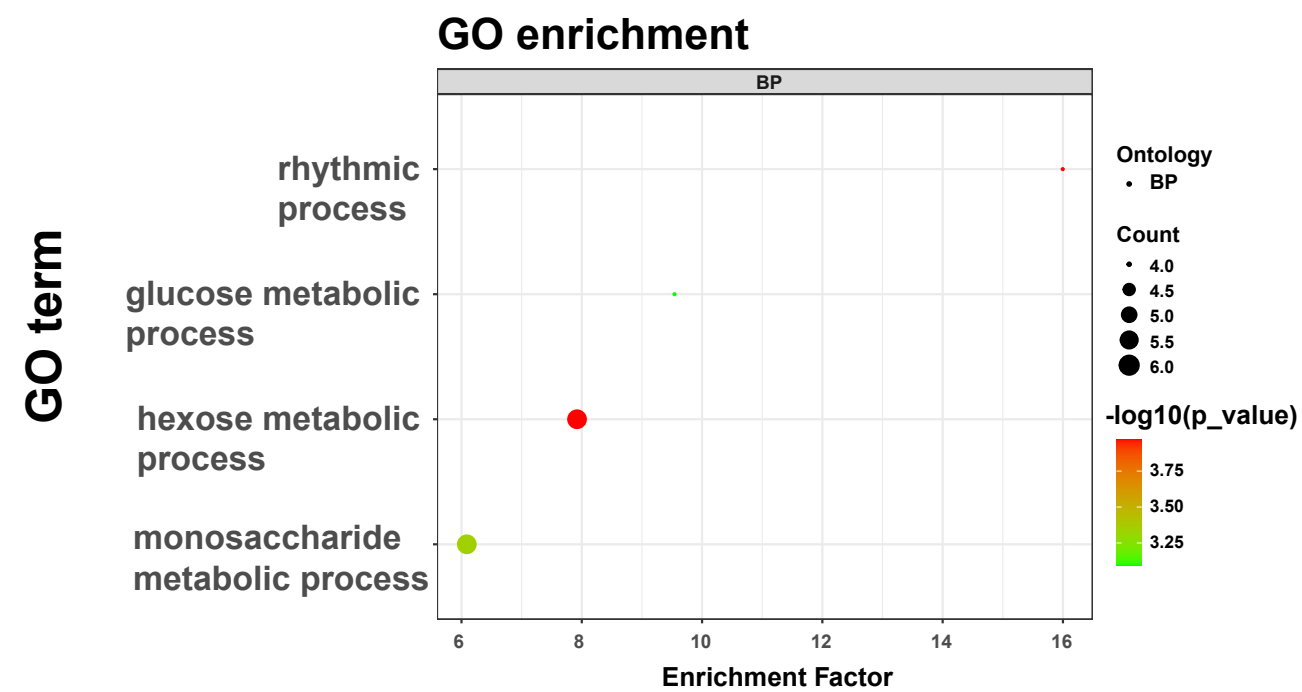

B

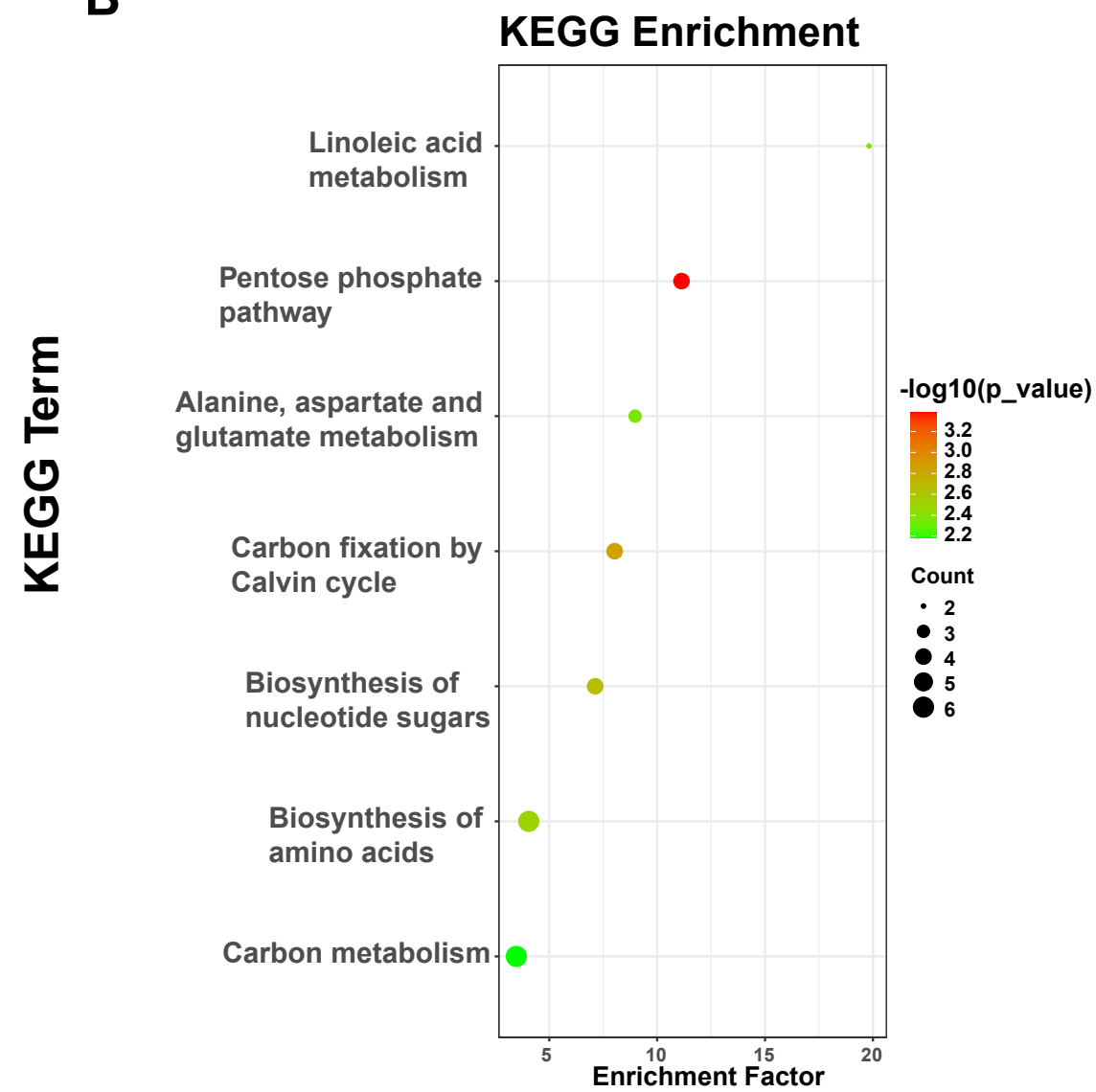

C

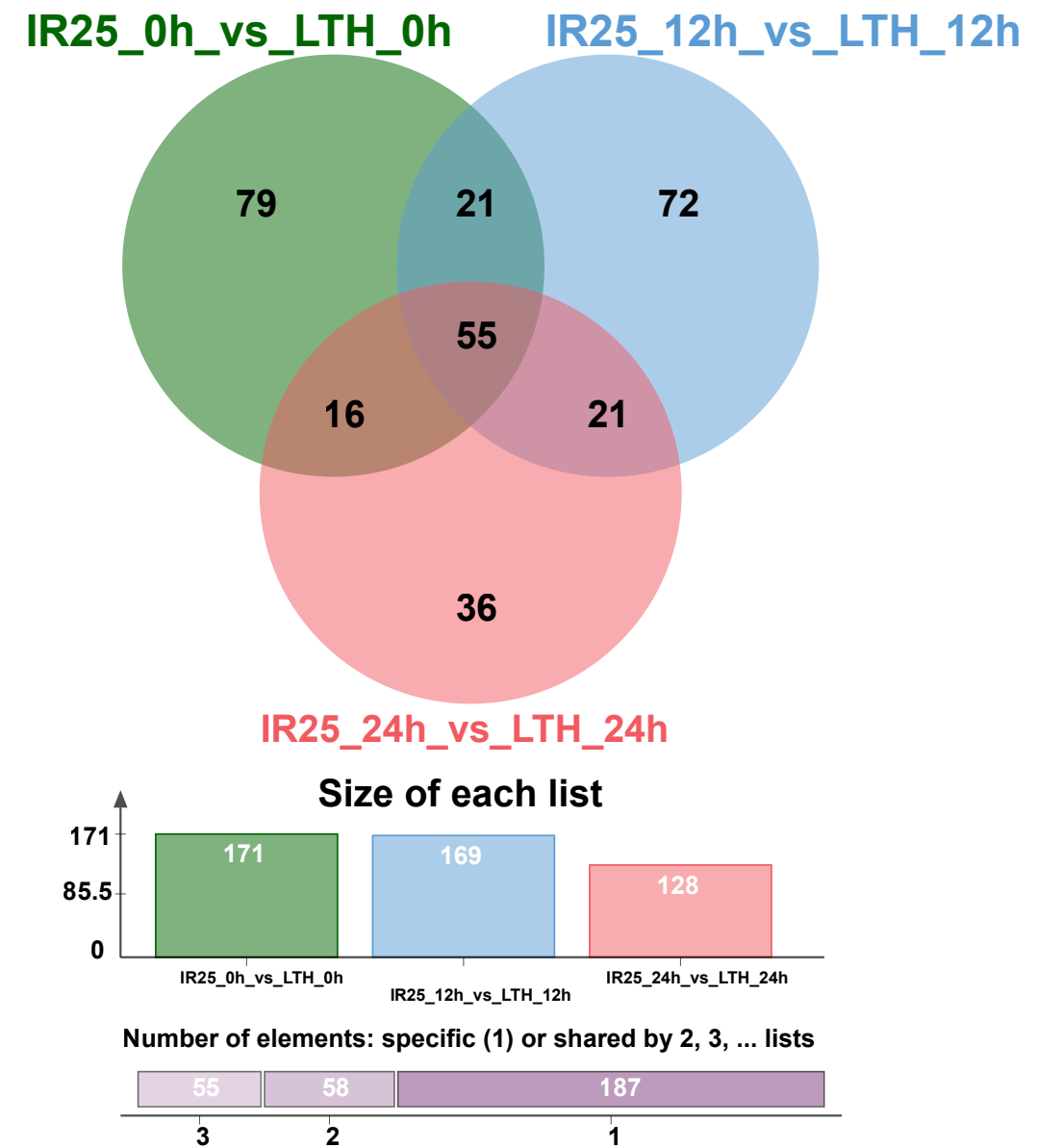

D

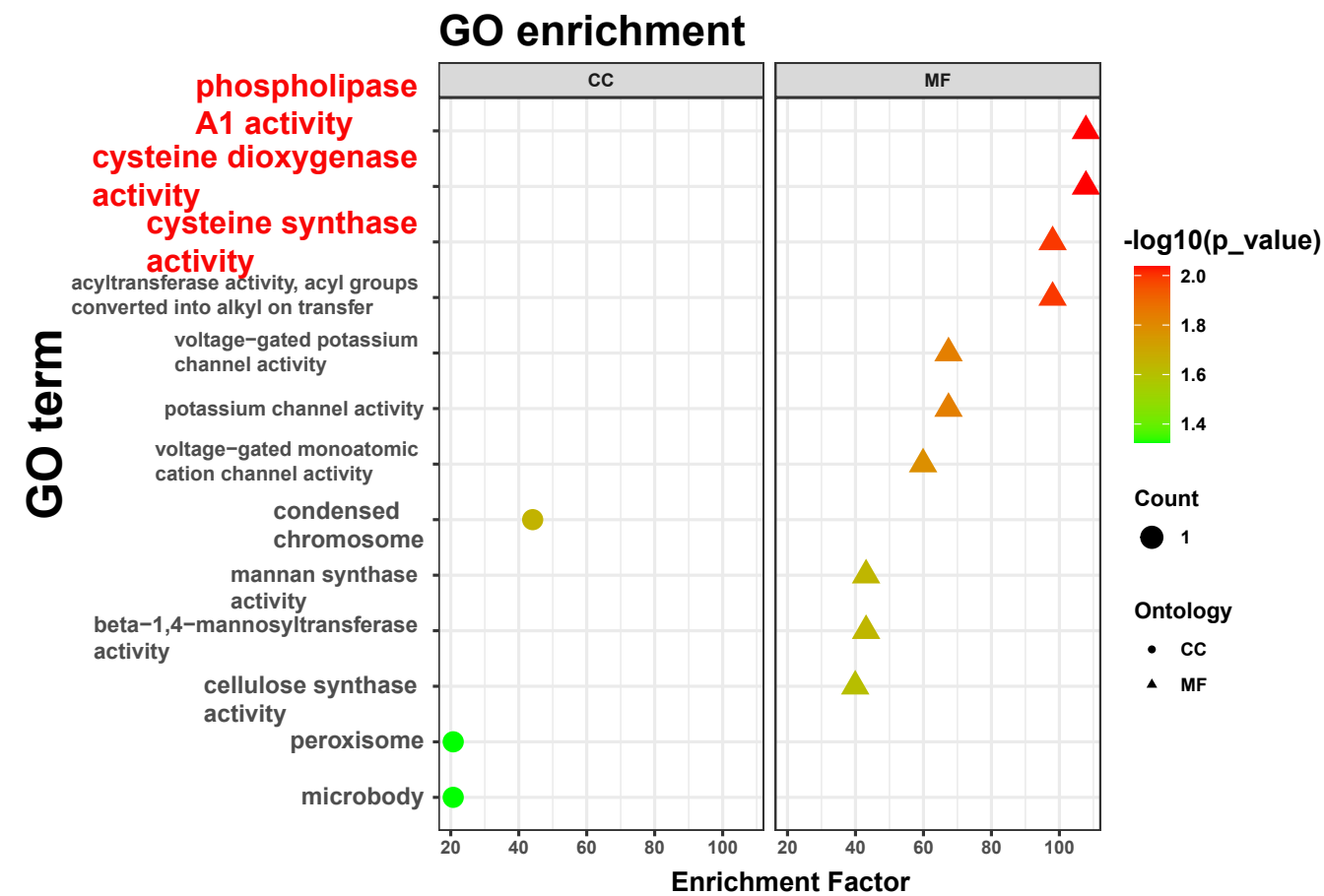

E

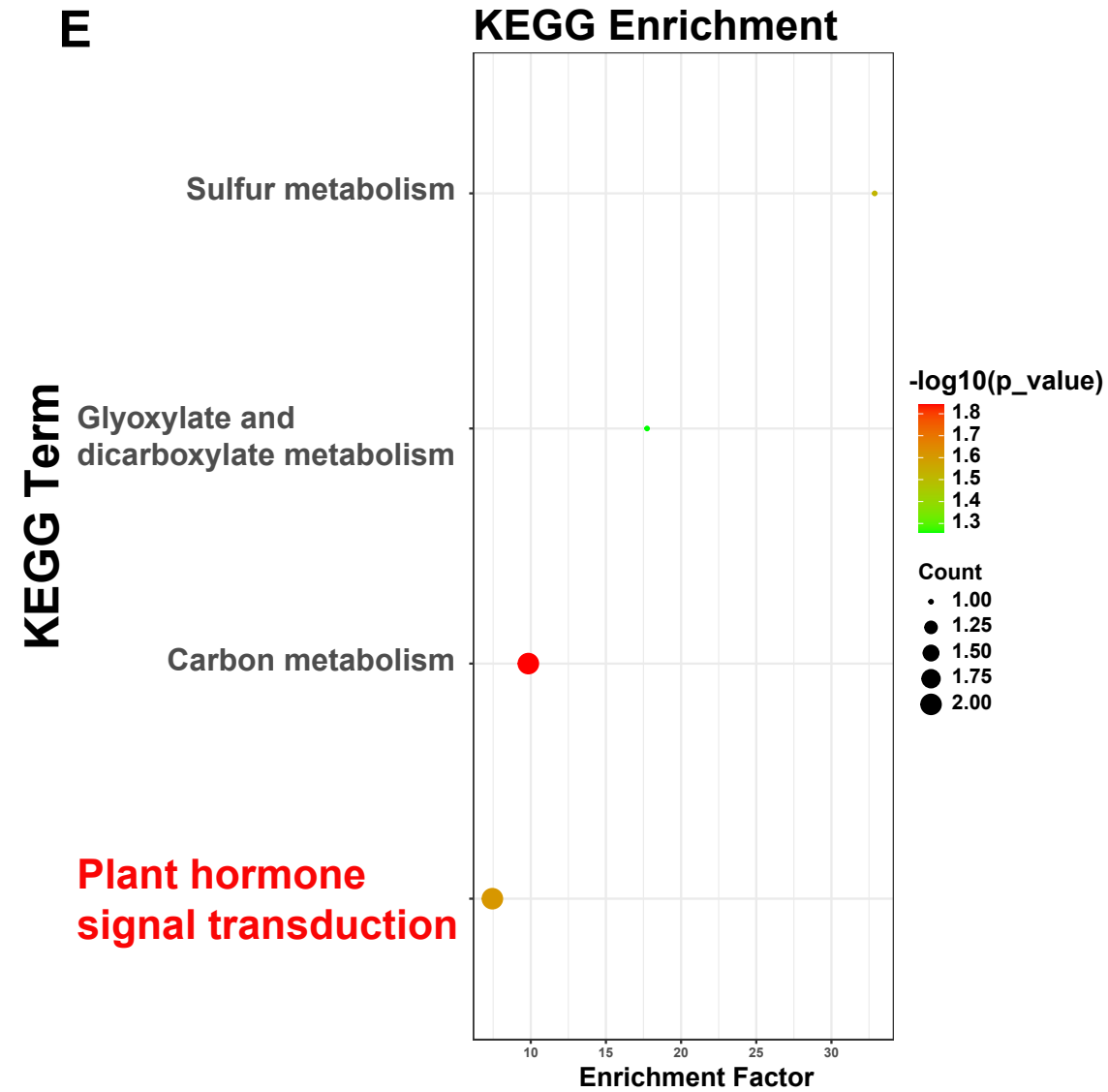

F

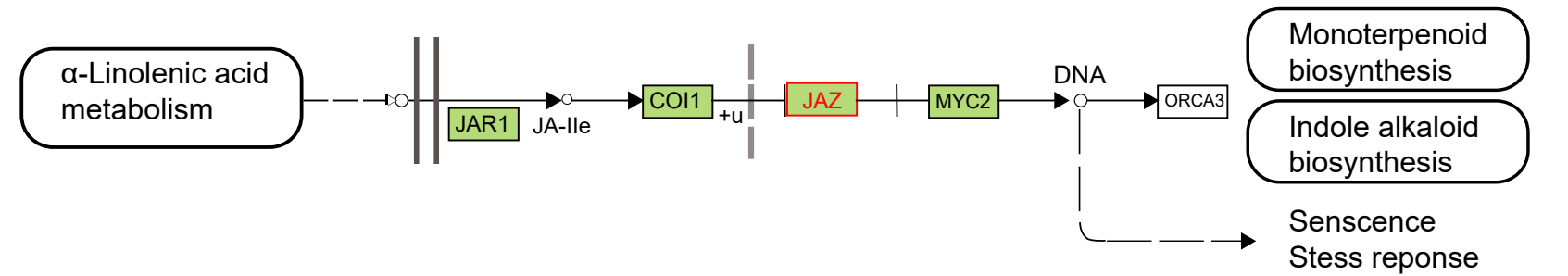

G

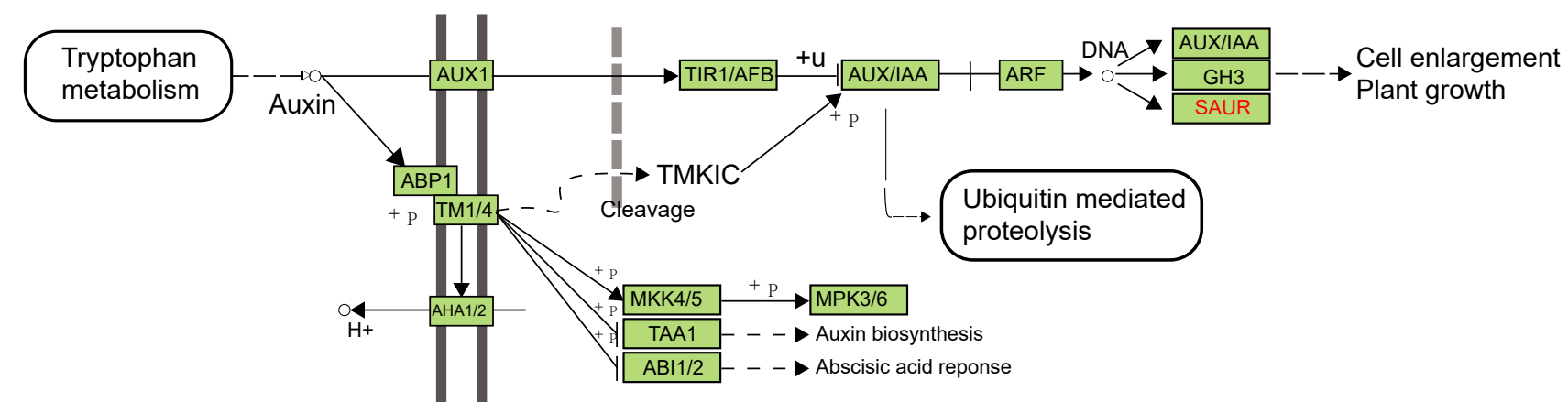

Supplement: Supplementary file 1 [file plants-14-02752-s001.zip › Figure S4.pdf]

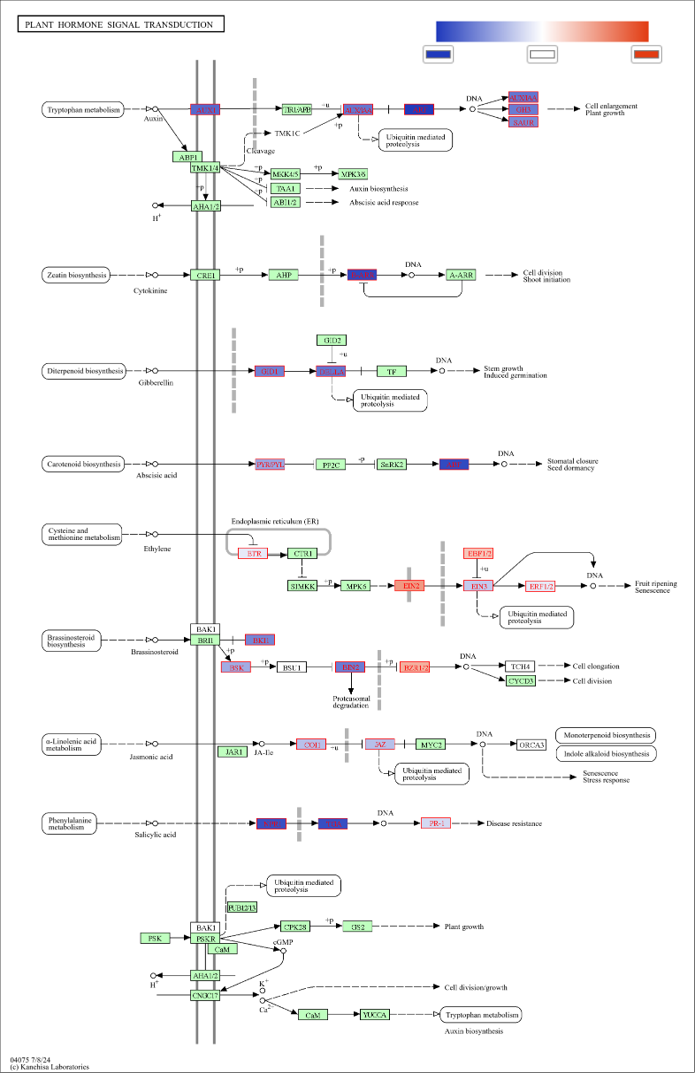

Supplement: Supplementary file 1 [file plants-14-02752-s001.zip › Figure S6.png]

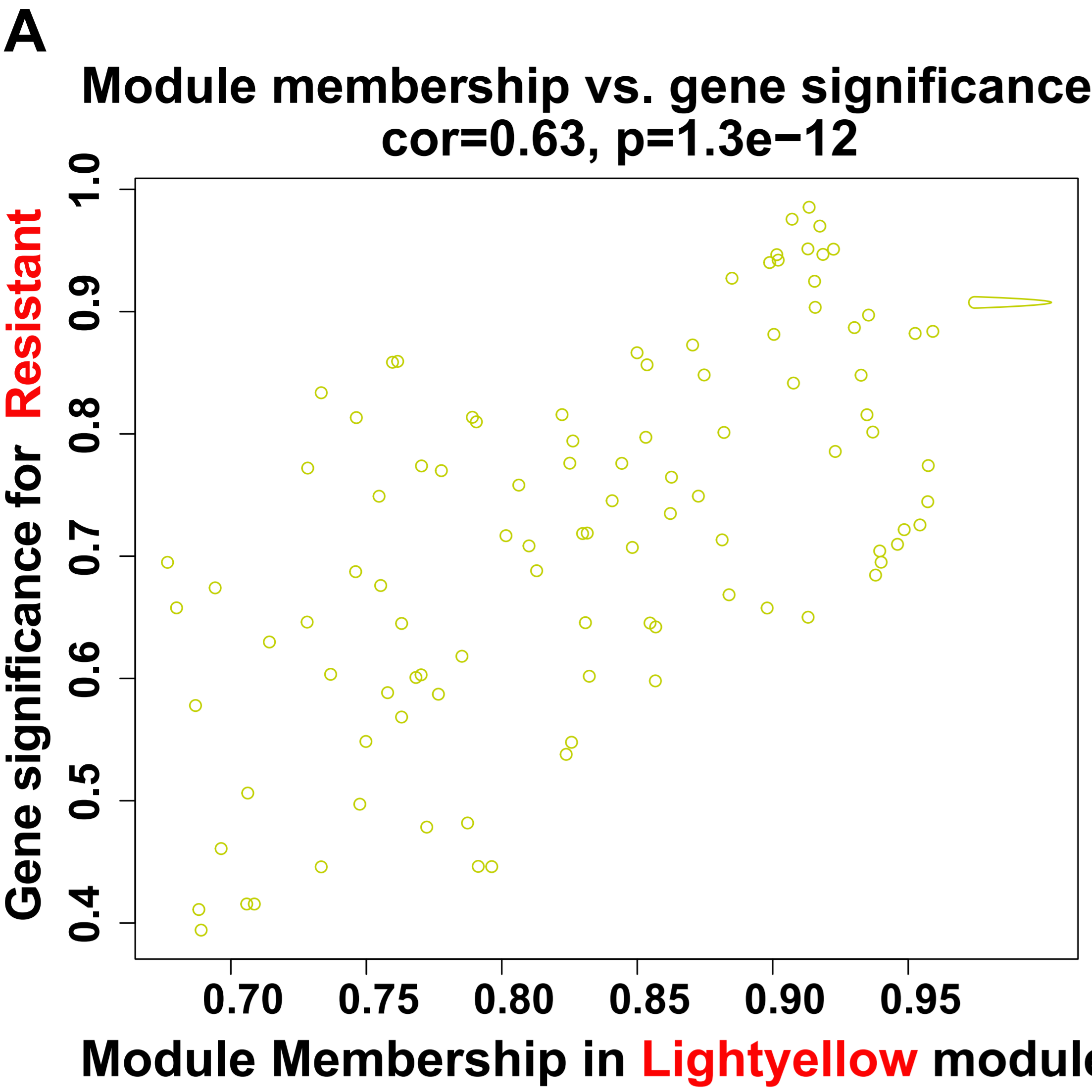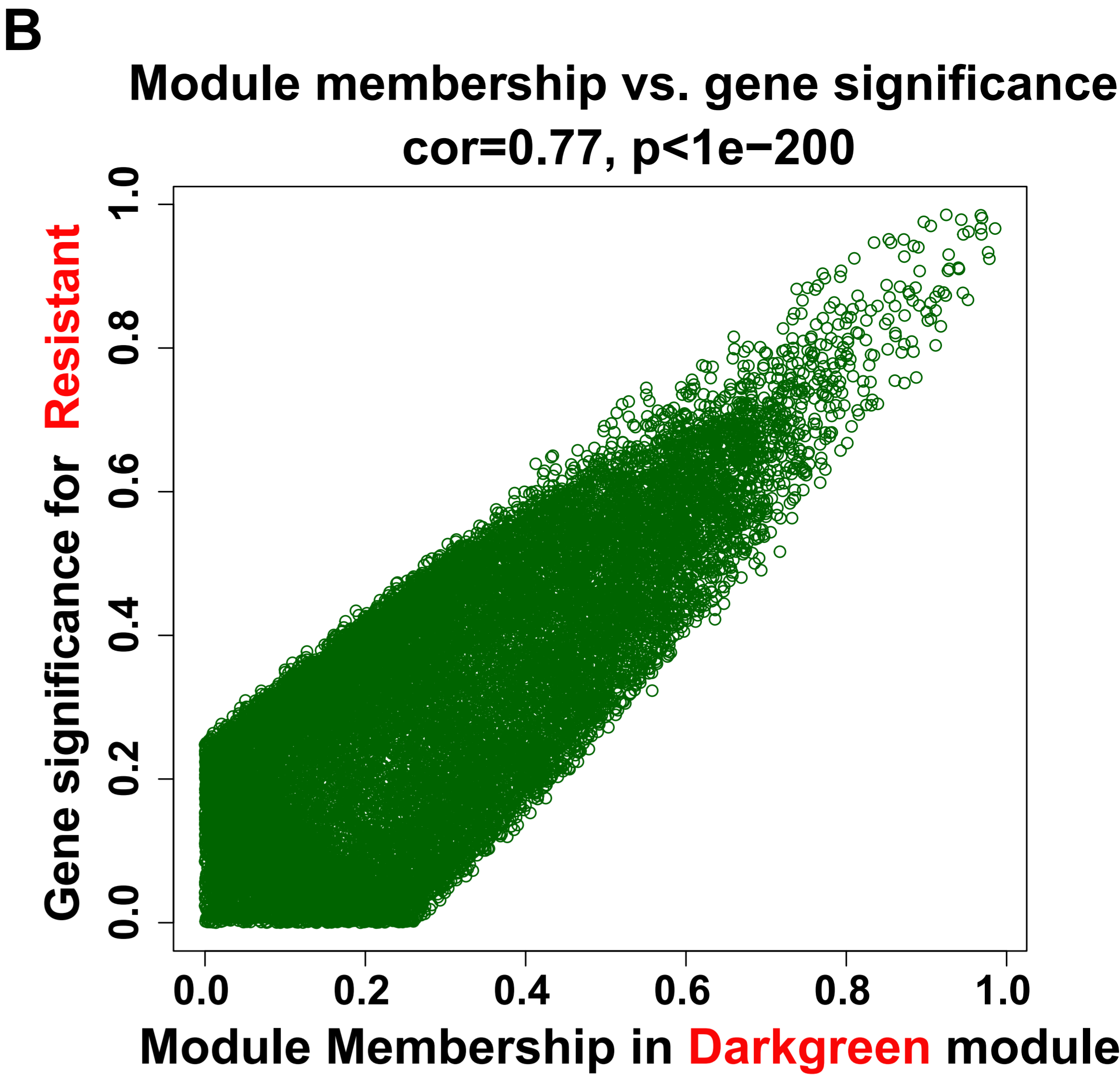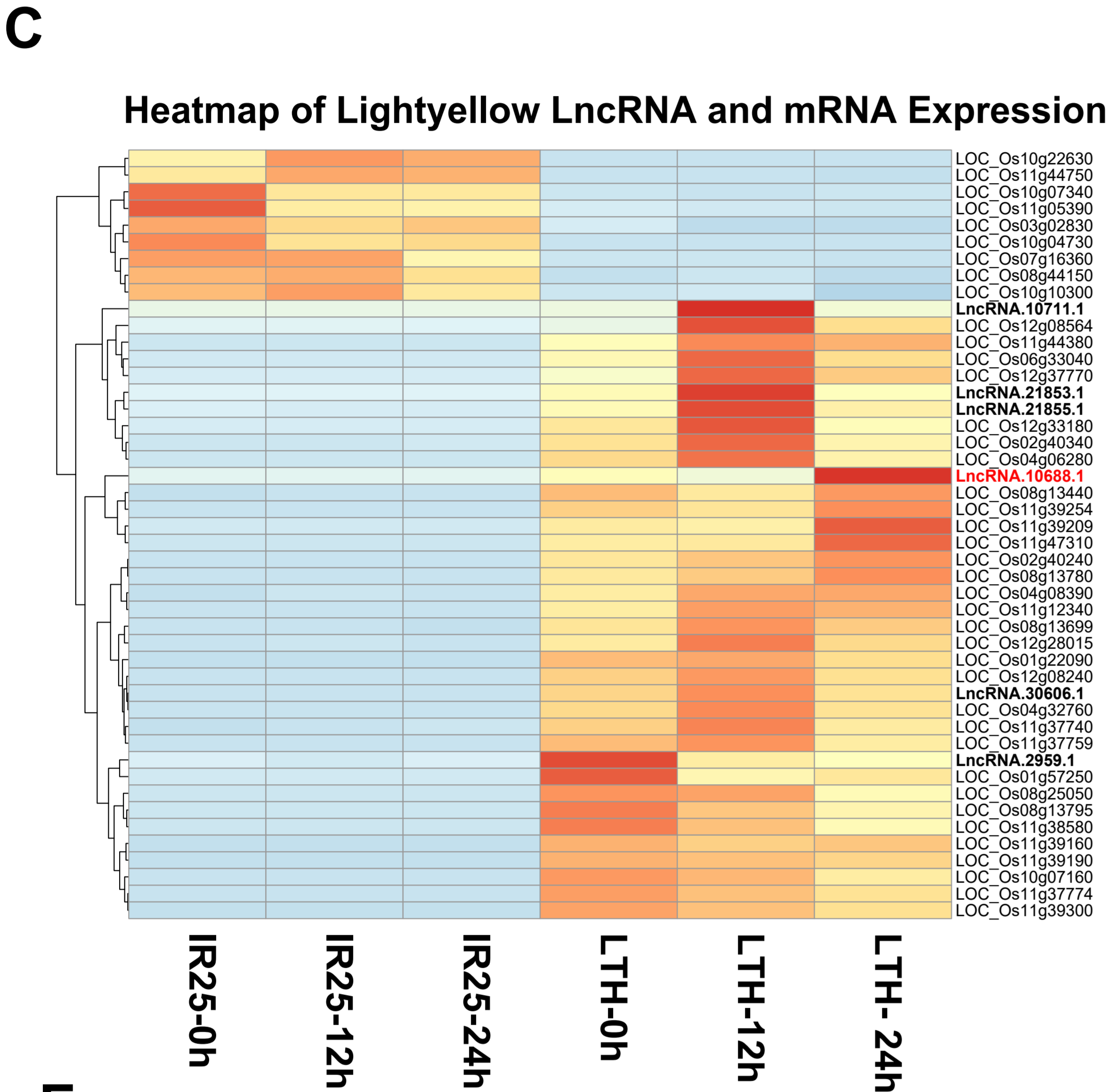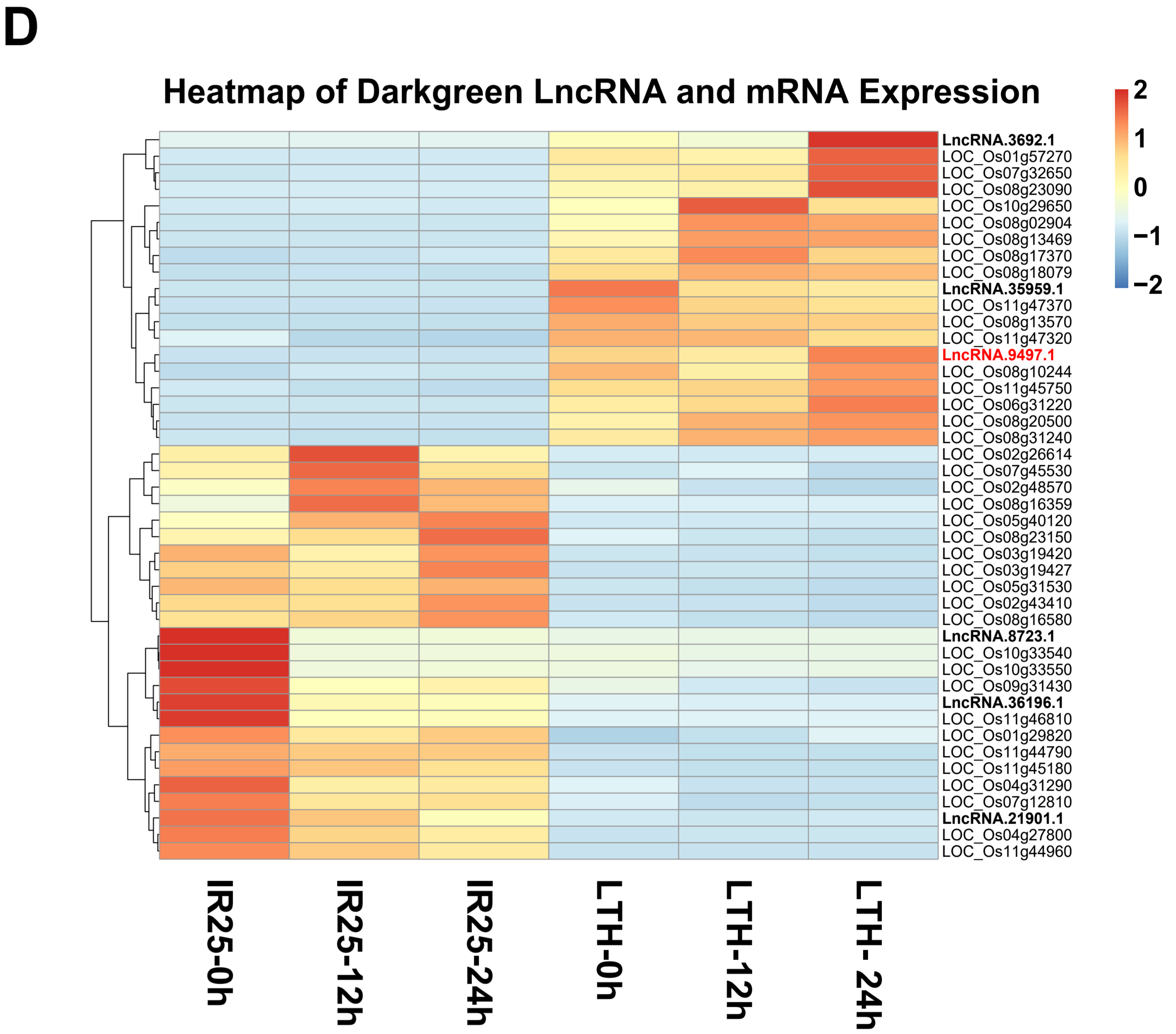

**Heatmap of Grey60 LncRNA and mRNA Expression**

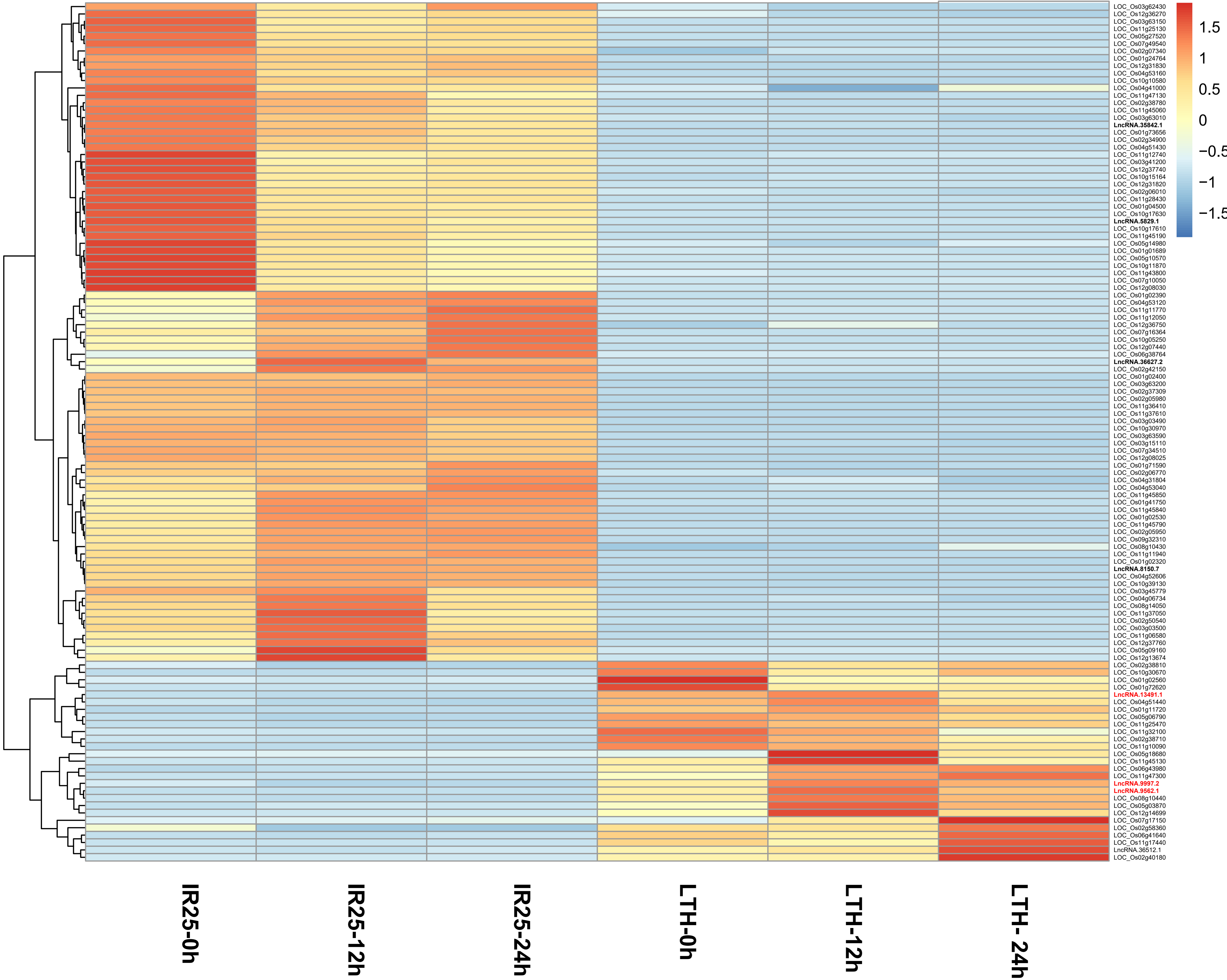

Supplement: Supplementary file 1 [file plants-14-02752-s001.zip › Figure S7.pdf]
